# GhPsbO breaks the growth-immunity tradeoff by simultaneously promoting growth and defense in cotton

**DOI:** 10.1093/plcell/koag190

**Published:** 2026-06-19

**Authors:** Jianing Li, Qiankun Wang, Hang Zhao, Ye Wang, Yanli Chen, Haodan Zhou, Hongying Duan, Lisen Liu, Fuguang Li, Xiaoyang Ge

**Affiliations:** State Key Laboratory of Cotton Bio-Breeding and Integrated Utilization, Institute of Cotton Research, Chinese Academy of Agricultural Sciences, Anyang 455000, China; State Key Laboratory of Cotton Bio-Breeding and Integrated Utilization, Institute of Cotton Research, Chinese Academy of Agricultural Sciences, Anyang 455000, China; College of Life Sciences, Henan Normal University, Xinxiang 453000, China; State Key Laboratory of Cotton Bio-Breeding and Integrated Utilization, Institute of Cotton Research, Chinese Academy of Agricultural Sciences, Anyang 455000, China; School of Life Sciences, Shandong Key Laboratory of Wetland Ecology and Biodiversity Conservation in the Lower Yellow River, Qufu Normal University, Qufu 273165, China; State Key Laboratory of Cotton Bio-Breeding and Integrated Utilization, Institute of Cotton Research, Chinese Academy of Agricultural Sciences, Anyang 455000, China; State Key Laboratory of Cotton Bio-Breeding and Integrated Utilization, Institute of Cotton Research, Chinese Academy of Agricultural Sciences, Anyang 455000, China; State Key Laboratory of Cotton Bio-Breeding and Integrated Utilization, Institute of Cotton Research, Chinese Academy of Agricultural Sciences, Anyang 455000, China; College of Life Sciences, Henan Normal University, Xinxiang 453000, China; State Key Laboratory of Cotton Bio-Breeding and Integrated Utilization, Institute of Cotton Research, Chinese Academy of Agricultural Sciences, Anyang 455000, China; State Key Laboratory of Cotton Bio-Breeding and Integrated Utilization, Institute of Cotton Research, Chinese Academy of Agricultural Sciences, Anyang 455000, China; State Key Laboratory of Cotton Bio-Breeding and Integrated Utilization, Institute of Cotton Research, Chinese Academy of Agricultural Sciences, Anyang 455000, China

## Abstract

The evolutionarily conserved tradeoff between pathogen resistance and crop yield remains a major bottleneck in agricultural breeding. Although numerous genes governing disease resistance or growth productivity have been identified, the pleiotropic regulatory factors that harmonize these 2 agronomic traits remain largely elusive. Here, we report that the cotton (*Gossypium hirsutum*) chloroplast oxygen-evolving complex protein GhPsbO is dually modulated at both translational and transcriptional levels by the *Verticillium dahliae* effector Vd10375. Overexpression of GhPsbO enhances photosynthesis and accelerates lignin deposition to establish structural defense, thereby simultaneously improving crop yield and disease resistance in cotton. Unexpectedly, knockout of GhPsbO enhanced resistance to *V. dahliae* by elevating chloroplast-derived reactive oxygen accumulation and result in plant sacrifices. Consistently, transcriptional repression of GhPsbO by overexpression of the transcription factor GhMYB44 significantly improved cotton resistance, mirroring the GhPsbO knockout disease-resistant phenotype. Collectively, our findings uncover a regulatory module balancing plant growth and immunity, and provide a promising molecular strategy for breaking the growth-defense tradeoff to achieve sustainable crop production.

## Teaser

Modulating a key chloroplast protein balances cotton growth and disease resistance, enhancing both photosynthesis and structural defense while revealing a route to overcome the growth-defense tradeoff.

## Introduction

Achieving concurrent improvement of yield and disease resistance has long posed a challenge in global crop breeding. While recent efforts have yielded notable theoretical and practical progress in enhancing disease resistance (Qi et al. 2019; Tirnaz and Batley 2019; Ma et al. 2021), primarily through the characterization of pathogen effectors and their host interactors, such as Vd06254–GhMYC3 in cotton and Hasp98–TaMAPK4 in wheat (Mahmood et al. 2022; Wei et al. 2023; Ma et al. 2025), immune activation often suppresses growth, resulting in yield penalties. This antagonism between defense and growth remains a central barrier to developing cultivars that perform optimally under both biotic stress and agronomic demand (He et al. 2022; Xu et al. 2022b). Consequently, breeding elite cotton varieties that integrate robust resistance without compromising yield has become a key objective (Zhao et al. 2023).

To address this tradeoff, 2 principal strategies have emerged. One involves identifying pleiotropic genes capable of regulating both traits. While a few such regulators (Wang et al. 2018; Qi et al. 2024), like the chloroplast elongation factors StTuA and StTuB in potato (Qi et al. 2024), exhibit dual functionality, many, including *IPA1* and *Pigm* in rice, promote resistance at the cost of growth, limiting their agronomic utility (Deng et al. 2017; Wang et al. 2018). The second strategy employs pathogen-inducible promoters to drive resistance genes, thereby restricting immune activation to infection periods and reducing fitness costs in uninfected conditions (Wang et al. 2018; Li et al. 2023). Although these approaches have shown partial success, neither fully resolves the intrinsic yield–defense antagonism. Thus, developing innovative strategies that can simultaneously enhance both traits remains a pressing challenge in modern crop improvement.

Chloroplasts, the sites of photosynthesis, are metabolic hubs that convert light energy into chemical energy and underpin the biomass foundation of agricultural productivity (Zhang et al. 2023). Accordingly, enhancing photosynthetic efficiency has become central to crop improvement strategies. Intriguingly, recent evidence reveals that chloroplast-localized proteins also contribute to immunity. For example, the pepper mosaic virus (PMMoV) coat protein interacts with chloroplast membrane protein OMP24, triggering ROS production to inhibit infection (Han et al. 2023). Similarly, StTuA, and StTuB, targeted by the *Phytophthora infestans* RXLR effector Pi22926, enhance both growth and immunity in potato (Qi et al. 2024). These findings suggest that pleiotropic chloroplast proteins may offer a paradigm for overcoming the growth–immunity tradeoff. However, despite this conceptual advance, few such proteins have been characterized, and their potential for dual-trait enhancement remains underexplored.

Cotton, a major global cash crop, is vital to the textile and food industries (Zhao et al. 2022; Qanmber et al. 2024). However, its production is threatened by *Verticillium* wilt, a devastating disease often termed the “cancer of cotton,” which causes severe yield losses and crop failure across key growing regions (Zhang et al. 2021, 2024b; Xu et al. 2022a). In this study, we identify GhPsbO, a chloroplast oxygen-evolving complex protein, as a key regulator of the yield–defense balance. Through pathogenomics screening, we discovered Vd10375, a virulence effector secreted by *V. dahliae* strain Vd991. Mechanistic analysis revealed that during host invasion, Vd10375 interacts directly with GhPsbO to stabilize its protein and represses the antisense lncRNA lncPsbO, leading to elevated GhPsbO accumulation in chloroplasts. The accumulated GhPsbO enhances disease resistance by promoting lignin biosynthesis and improves photosynthetic output, thereby increasing yield. Additionally, we found that the transcription factor GhMYB44 represses *GhPsbO* expression during infection, fine-tuning ROS-mediated immunity to maintain homeostasis. Together, these findings reveal a previously uncharacterized Vd10375–lncPsbO–GhPsbO regulatory circuit that integrates photosynthesis and immune signaling. Our work offers a promising strategy for overcoming the yield–defense tradeoff in cotton and provides a molecular foundation for precision breeding of high-performance cultivars.

## Results

### Vd10375 triggers plant cell death and contributes to *V. dahliae* virulence in cotton

**Figure. 1.**
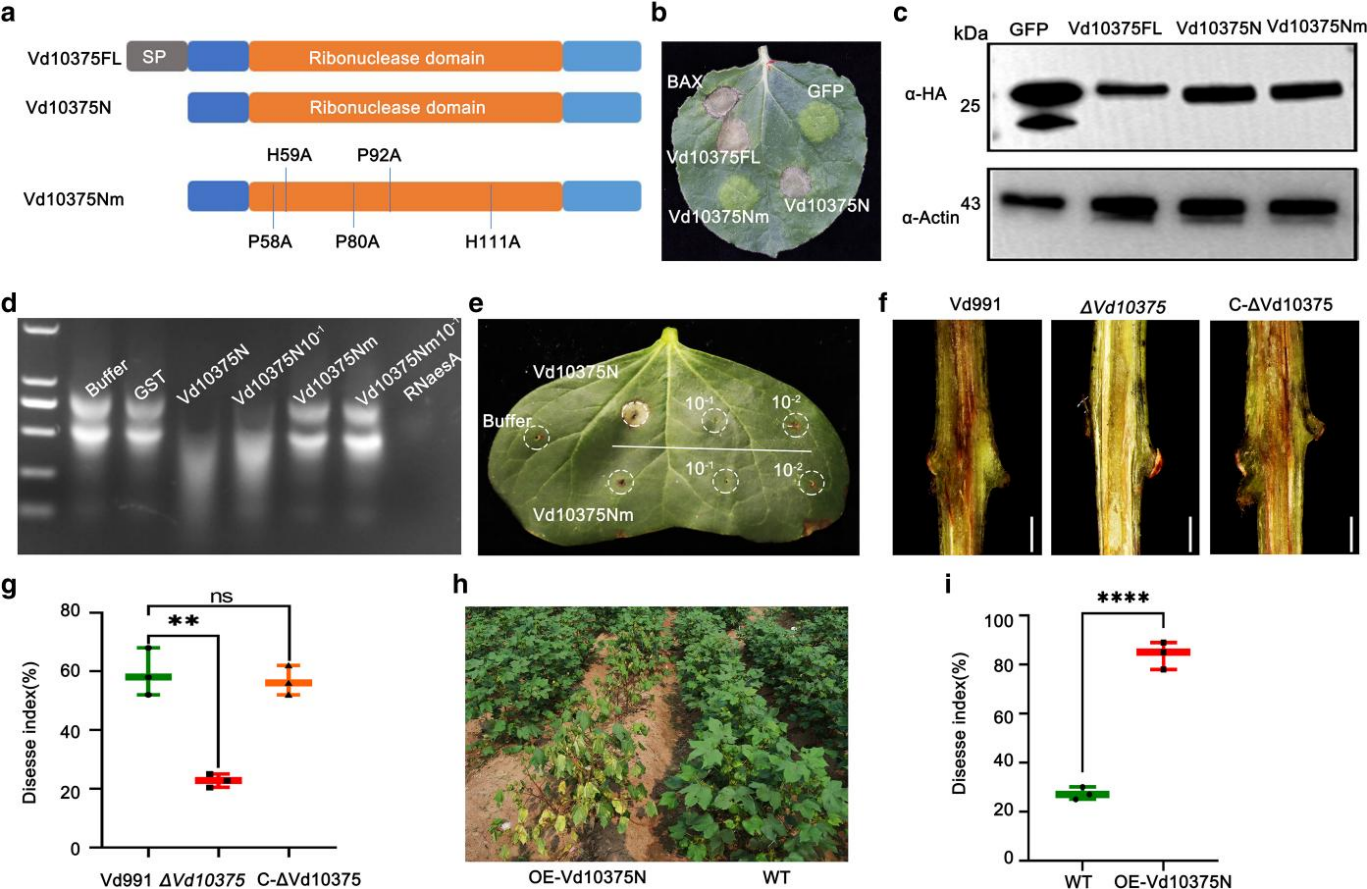
Vd10375 triggers cell death and enhances *V. dahliae* virulence in cotton. a) Schematic representation of Vd10375 constructs: the full-length protein (containing the signal peptide, SP), Vd10375N (full-length without SP), and Vd10375Nm (with catalytic site mutations). b) Transient expression in *Nicotiana benthamiana* leaves via *Agrobacterium tumefaciens* infiltration demonstrated that Vd10375FL and Vd10375N induce visible cell death, whereas Vd10375Nm does not. BAX and GFP were used as positive and negative controls, respectively. Photographs were taken 5 d postinfiltration. c). Immunoblot analysis confirmed the expression of 3×HA-tagged Vd10375FL, Vd10375N, and Vd10375Nm in *N. benthamiana* leaves. Actin served as a loading control. d) RNase activity assay using recombinant proteins expressed in *E. coli* showed that Vd10375N (1 μM) degrades total RNA extracted from cotton, whereas Vd10375Nm lacks activity. RNase A was used as a positive control, while GST and buffer served as negative controls. Reactions were incubated for 10 min. e) Cotton leaves infiltrated with purified Vd10375N and Vd10375Nm proteins (0.002 to 0.2 μg) exhibited cell death only in the presence of Vd10375N. Images were taken at 3 d postinfiltration. f) Disease symptoms in the susceptible cotton cultivar JM11 at 24 d postinoculation (dpi) with wild-type *V. dahliae* Vd991, ΔVd10375 mutant, and complemented ΔVd10375 strains. Representative images from 3 biological replicates are shown. Scale bar = 2 mm. g) Quantitative assessment of disease severity using a disease index (DI), calculated as: DI = 100% × (∑ representative level × number of diseased leaves at each level)/(total number of investigated leaves × 4). Five severity levels (0, 1, 2, 3, and 4) were used (Error bars represent ± standard error (SE); student's *t*-test: **P < 0.01). Each data point represents the mean disease index from 3 independent experiments containing 30 plants in total for each treatment. h to i) Field evaluation of wild-type and transgenic cotton plants overexpressing Vd10375N (OE-Vd10375N). Representative symptoms (h) and corresponding DI values (i) demonstrate enhanced disease susceptibility in transgenic lines (student's *t*-test: ****P < 0.0001). Each data point represents the mean disease index from 3 independent experiments containing 30 plants in total for each treatment.

Previous secretome analyses identified 739 predicted secreted proteins encoded by *V. dahliae* strain Vd991 (Yin et al. 2022), though only a few have been functionally characterized for their roles in host immunity modulation. To explore candidate effectors, we screened 20 secreted proteins containing signal peptides (SPs), each was cloned into the PVX vector pGR107 (Table S1), for their ability to induce cell death following transient expression in *Nicotiana benthamiana*. At 7 d postinfiltration, Vd10375 (UniProt ID: G2WRC4), an RNase-encoding effector, triggered marked cell death. UniProt analysis identified 5 conserved residues (P58, H59, P80, P92, H111) essential for RNase activity (Figure S1, Fig. 1a) To validate its secretory function, we employed a signal sequence trap system and confirmed SP activity via invertase enzymatic assay (Figure S2b). To determine the functional domains of Vd10375 required for its cell death induction activity, we generated a series of Vd10375 deletion mutants and analyzed them by the way of cell death assays. The SP-deletion variant retained its ability to induce cell death (Fig. 1b), suggesting that the SP was dispensable for this activity.

A catalytically inactive mutant (Vd10375Nm: P58A/H59A/P80A/P92A/H111A) was generated, and *Agrobacterium*-mediated transient expression of Vd10375Nm in *N. benthamiana* confirmed its inability to induce cell death (Fig. 1, b and c). RNase assays with cotton total RNA revealed that Vd10375N degraded RNA in a dose-dependent manner within 5 min, whereas Vd10375Nm failed to exhibit any activity (Fig. 1d), a phenotype also observed in *Gossypium hirsutum* cotyledons following infiltration with purified protein (Fig. 1e). Together, these results demonstrate that Vd10375N-induced cell death depends on its catalytic RNase activity.

To further assess its virulence function, we generated *ΔVd10375* knockout mutants by replacing the coding region with a hygromycin resistance cassette, and corresponding complementation strains (C-ΔVd10375) by reintroducing Vd10375 under its native promoter. PCR validated 2 independent lines of each genotype (Figure S2c). Virulence assays revealed that *ΔVd10375* mutants exhibited markedly reduced disease symptoms—including less leaf necrosis, milder wilting, and diminished vascular damage—compared to wild-type Vd991 and complemented strains (Fig. 1, f and g), confirming Vd10375 as a key virulence effector. Schematic diagram of symptoms used to determine disease index (DI) in Figure S2d, corresponding to each of the 5 disease severity grades (Grade 0: Asymptomatic; Grade 1: One true leaf turns yellow; Grade 2: Two true leaves show obvious wilting; Grade 3: Three or more leaves wilt and dry up; Grade 4: Plant death). To evaluate the pathogenic potential of Vd10375, we generated transgenic cotton lines overexpressing Vd10375N (*OE-Vd10375N*), with confirmed protein expression (Figure S2e). Both field trials and greenhouse showed that *OE-Vd10375N* plants exhibited heightened susceptibility to *V. dahliae*, with significantly elevated disease indices compared to wild-type controls (Fig. 1, h and i; Figure S2, f and g).

### Vd10375 interacts with GhPsbO in vitro and in vivo

**Figure. 2.**
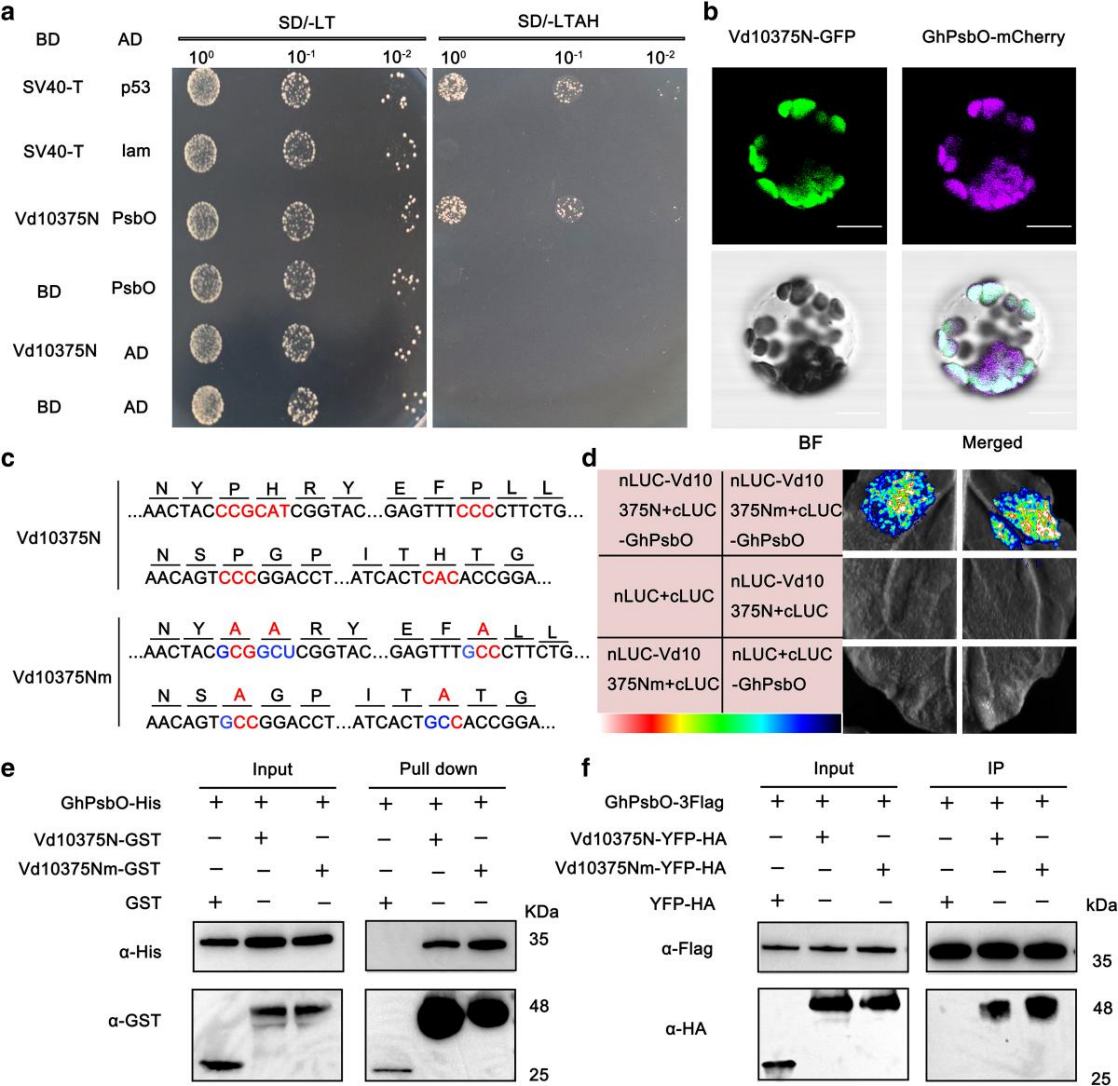
Vd10375 directly interacts with GhPsbO in vitro and in vivo. a) Yeast 2-hybrid (Y2H) assay confirmed the interaction between Vd10375N and GhPsbO. pGBKT7-Vd10375N and pGADT7-GhPsbO were co-transformed into yeast strain AH109 and selected on SD/-Leu/-Trp/-His/-Ade medium. Plates were photographed after 4 d. Results were consistent across 3 biological replicates. b) Co-localization of Vd10375N-GFP and GhPsbO-mCherry in *Arabidopsis* protoplasts, 48 hours post-transformation. Confocal microscopy confirmed overlapping signals. Scale bar = 10 μm. c) Schematic highlighting mutation sites in Vd10375Nm, The upper part shows the wild-type sequence, and the lower part correspondingly shows the mutant sequence. d). Luciferase complementation imaging (LCI) assay in *N. benthamiana* leaves confirmed protein–protein interaction. Constructs expressing nLUC-Vd10375N or nLUC-Vd10375Nm and cLUC-GhPsbO were co-infiltrated. LUC signal was detected at 48 h postinfiltration. Negative controls included various noninteracting construct pairs. e) GST pull-down assay demonstrated direct binding between GhPsbO and both Vd10375N and Vd10375Nm recombinant proteins in vitro. f) Co-immunoprecipitation (Co-IP) assay confirmed in vivo interaction. Total proteins from *N. benthamiana* leaves co-expressing GhPsbO-3×Flag with either YFP-HA, Vd10375N-YFP-HA, or Vd10375Nm-YFP-HA were immunoprecipitated using anti-Flag beads. Western blot analysis with anti-HA antibody confirmed co-precipitation.

To elucidate the molecular basis of Vd10375N-mediated defense modulation, we performed a yeast 2-hybrid (Y2H) screen using a cotton cDNA library (Table S2). The majority of positive clones encoded GhPsbO, prompting further analysis. Y2H assays confirmed a strong interaction between Vd10375N and GhPsbO (Fig. 2a). Co-localization in *Arabidopsis* protoplasts indicated that the interaction occurs specifically in chloroplasts (Fig. 2b), which was further corroborated by luciferase complementation imaging (LCI), pull-down, and co-immunoprecipitation (Co-IP) assays (Fig. 2, d to f). Further subcellular localization analysis implied the localization of Vd10375N in both the chloroplast and nucleus (Figure S3), which supports its interaction with the chloroplast-localized protein GhPsbO. To determine whether enzymatic activity influences this interaction, we assessed the binding affinity of GhPsbO to Vd10375N and its catalytically inactive variant Vd10375Nm. LCI, pull-down, and Co-IP analyses consistently showed that both variants interact with GhPsbO (Fig. 2, c to f), indicating that the GhPsbO–Vd10375N interaction is independent of RNase active domain.

### Overexpression of *GhPsbO* simultaneously enhances disease resistance and yield in cotton

**Figure. 3.**
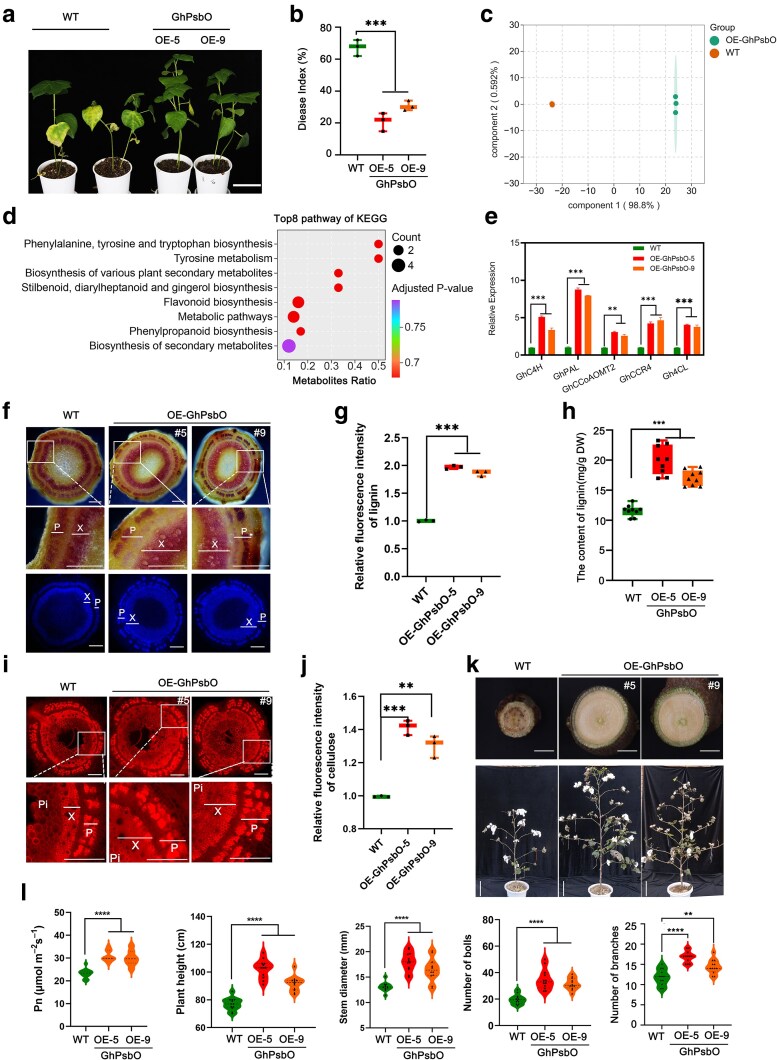
Overexpression of *GhPsbO* enhances cotton growth and immunity. a) Disease symptoms in wild-type (WT) and GhPsbO-overexpressing (*OE-GhPsbO*) cotton plants following *V. dahliae* infection at 28 dpi. Scale bar = 5 cm. b) Disease index quantification revealed significantly reduced symptoms in *OE-GhPsbO* lines compared to WT. (Error bars represent ± standard error (SE); student's *t*-test: ***P < 0.001). Each data point represents the mean disease index from 3 independent experiments containing 30 plants in total for each treatment. c) The partial least squares-discriminant analysis (PLS-DA) score plot showed a clear separation between the *OE-GhPsbO* group and the WT group, with strong homogeneity within each group. d) KEGG enrichment bubble plot from metabolomics analysis. The Rich factor is defined as the ratio of the number of differentially flavonoid metabolites annotated within a specific functional category to the total number of metabolites in that category, reflecting the relative enrichment of differential metabolites in lignin biosynthesis-related pathways. f.) Quantitative RT-PCR analysis of important genes involved in lignin biosynthesis pathway. Error bars mean ± standard deviation (SD) and stars indicate the statistical significance (student's *t*-test: **P-value < 0.01, ***P-value<0.001). Each data point represents the mean of 3 independent experiments in which each experiment contained 3 replicates. The error bars represent s.e.m. (*n* = 3). f) Lignin deposition was visualized in stem sections using resorcinol staining (top) and lignin autofluorescence (bottom). OE-GhPsbO plants showed greater lignification under both conditions. Tissues are labeled as: *P* = phloem; X = xylem; Pi = pith. g) Results of ImageJ software analysis of the lignin fluorescence intensity (Student's *t*-test, ***P-value < 0.001). Each data point represents the mean of 3 independent experiments in which each experiment contained 3 replicates. The error bars represent s.e.m. (*n* = 3). h) Lignin content in stems of transgenic lines overexpressing GhPsbO relative to control plants (Student's *t*-test, ***P-value < 0.001). Each data point represents an averaged value from 3 biological replicates with error bars indicating standard error. (*n* = 9) i) Pontamine Fast Scarlet 4B (S4B) staining showed enhanced cellulose deposition in roots of OE-GhPsbO plants 14 d after infection (dpi) with *Verticillium dahliae*. j) Results of ImageJ software analysis of the cellulose fluorescence intensity (Student's *t*-test, **P-value < 0.01, ***P-value < 0.001). Each data point represents the mean of 3 independent experiments in which each experiment contained 3 replicates. The error bars represent s.e.m. (*n* = 3). k) Morphological assessment of WT and *OE-GhPsbO* lines (*OE-5*, *OE-9*) grown under field conditions. Stem cross-sections indicate increased diameter in transgenic plants. Field plants were cultivated in disease nurseries, representative plants were transferred to pots for photography. Scale bar: upper 10 mm and lower 3 cm. l) Agronomic traits, including the net photosynthetic rate (Pn), plant height, stem thickness conducted on 90-d-old plants. Additionally, cotton plants at 140 d after planting were used to assess the number of fruiting branches and bolls. (Student's *t*-test, **P-value < 0.01, ****P-value < 0.0001). Each data point represents an averaged value from 3 biological replicates with error bars indicating standard error. (*n* = 12).

To examine GhPsbO's role in cotton immunity, we generated transgenic lines overexpressing *GhPsbO*, including *OE-GhPsbO-5* and *OE-GhPsbO-9*, with transcript levels elevated approximately 337-fold and 25-fold, respectively (Figure S4a). Upon *V. dahliae* inoculation, both lines displayed significantly enhanced resistance (Fig. 3, a and b), along with more developed root systems (Figure S4, b and c). To elucidate the mechanism by which GhPsbO enhances disease resistance in cotton, we conducted a metabolomic analysis of *OE-GhPsbO* and WT plants under *V. dahliae* stress conditions. The PLS-DA plot demonstrates a clear separation of the major differential metabolites between the overexpression and wild-type strains (Fig. 3c). KEGG analysis of significantly differentially accumulated metabolites revealed their significant enrichment in the biosynthesis of phenylalanine, a precursor of lignin synthesis. (Figure 3d), suggesting that overexpression of *GhPsbO* may increases lignin accumulation. We subsequently measured lignin content and examined the expression of lignin biosynthesis-related genes in both *OE-GhPsbO* and WT plants. The results showed that *OE-GhPsbO* plants accumulated higher levels of lignin and exhibited significantly elevated expression of key lignin synthesis genes compared to the wild-type controls (Fig. 3, e—to g). We further measured the content of lignin, which was significantly higher in *OE-GhPsbO* plants (Fig. 3h) Histochemical staining of *OE-GhPsbO* roots showed elevated cellulose deposition via Pontamine Fast Scarlet 4B (S4B) staining (Fig. 3, i and j). Using fluorescence in situ hybridization, we detected the expression of *GhPsbO* predominantly in cells associated with phloem and xylem in the root, reinforcing its contribution to root lignification and pathogen resistance (Figure S4d). To further investigate whether PsbO contributes to disease resistance by modulating lignin content, we silenced the lignin biosynthesis gene GhPAL in *OE-GhPsbO* plants. The resulting transgenic lines exhibited a partial reversion of the disease-resistant phenotype observed in the *OE-GhPsbO* background, displaying clear susceptibility traits (Figure S4, e—to g). Transverse stem sections from field-grown OE lines revealed reduced lesion areas and increased stem thickness compared to wild type, supporting GhPsbO's role in disease resistance and agronomic performance (Fig. 3k).

Given the established roles of chloroplast-localized proteins in optimizing photosynthesis and yield (Wang et al. 2023; Chen et al. 2024; Salesse-Smith et al. 2025), we hypothesized that GhPsbO may also enhance biomass production. Field trials demonstrated significantly increased net photosynthetic rate (Pn), plant height, stem diameter, fruiting branch number, and boll count in OE-*GhPsbO* lines compared with WT controls (Fig. 3l). These findings collectively establish GhPsbO as a dual-function regulator that simultaneously improves yield and disease resistance, representing a promising molecular target for cotton breeding.

### Vd10375 inhibits the degradation of GhPsbO

**Figure. 4:**
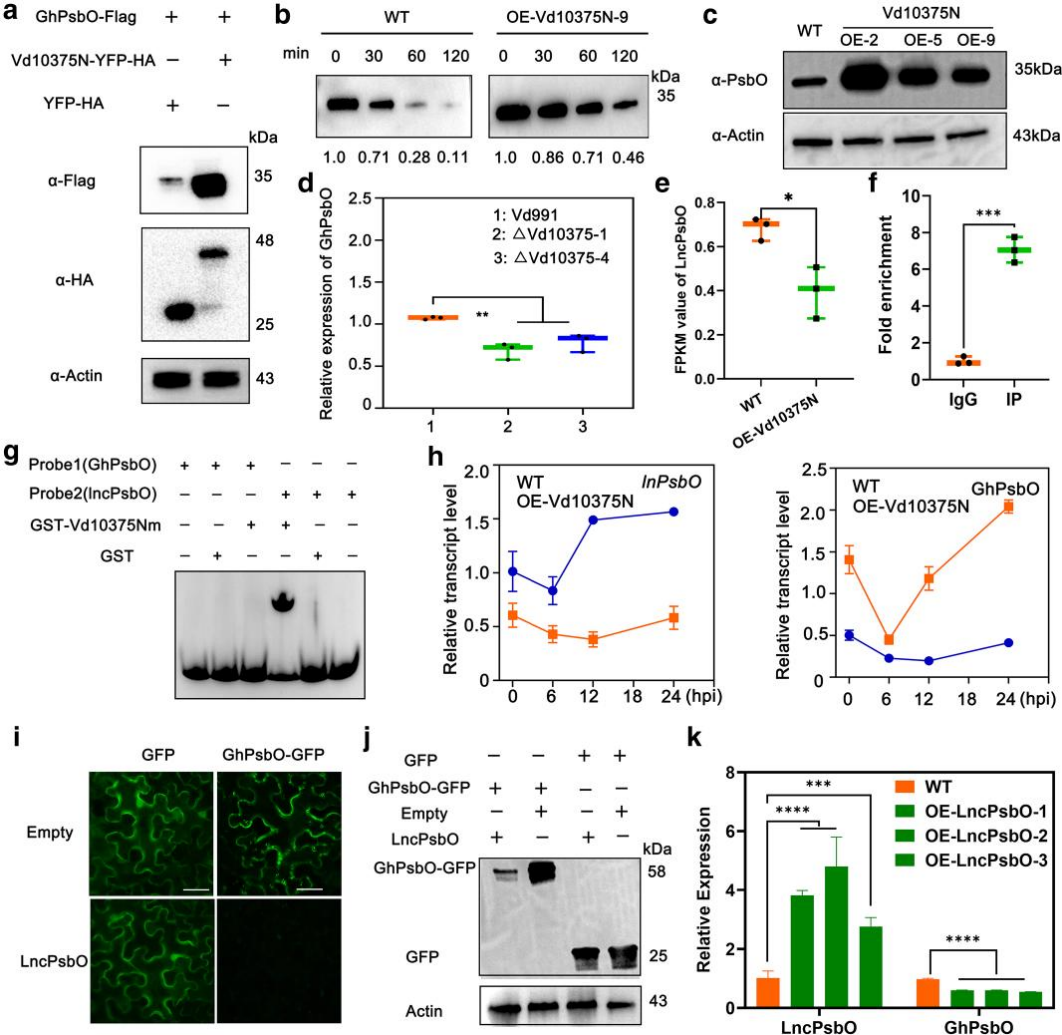
Vd10375 inhibits GhPsbO degradation via post-transcriptional and transcriptional regulation. a) Co-expression of Vd10375N enhanced GhPsbO protein accumulation in *Nicotiana benthamiana*. Leaves were co-infiltrated with *Agrobacterium tumefaciens* carrying GhPsbO-3×Flag and either Vd10375N-HA or YFP-HA (control). Immunoblotting using anti-HA and anti-Flag antibodies detected the respective proteins, with actin serving as a loading control. b) In vitro degradation assay of purified recombinant GhPsbO-His protein co-incubated with total protein extracts from wild-type (WT) and OE-*Vd10375N-9* cotton plants for 0, 30, 60, and 120 min. Degradation was reduced in the presence of Vd10375N, suggesting its stabilizing effect on GhPsbO. Numbers below the immunoblot image represent quantification normalized to the 0 min control. c) Immunoblot analysis of endogenous GhPsbO levels in WT and 3 independent *OE-Vd10375N* cotton lines showed that Vd10375N promotes GhPsbO accumulation *in planta*. Actin was used as the loading control. d) Quantitative RT-PCR analysis revealed that GhPsbO transcript levels were reduced in cotton roots infected with the ΔVd10375 mutant compared to the wild-type. (Student's *t*-test, **P < 0.01). Each data point represents the mean value of 3 independent biological replicates per treatment (mean ± SE), error bars represent ± SE of the dataset. e) Expression analysis of the antisense long noncoding RNA *lncPsbO* showed its suppression in *OE-Vd10375N* lines, indicating that Vd10375N may promote *GhPsbO* expression by downregulating *lncPsbO* (Student's *t*-test, *P < 0.05). Each data point represents the mean value of 3 independent biological replicates per treatment (mean ± SE), error bars represent ± SE of the dataset. f) RNA immunoprecipitation (RIP-qPCR) assay using an anti-HA antibody demonstrated direct association between Vd10375N and *lncPsbO* RNA *in planta*. Mouse IgG served as the control. Data represent means ± SE from 3 biological replicates (Student's *t*-test, ***P < 0.001). Each data point represents the mean value of 3 independent biological replicates per treatment (mean ± SE), error bars represent ± SE of the dataset. g) In vitro binding between Vd10375Nm and *GhPsbO* or *lncPsbO* RNA by RNA EMSA. The indicated biotinylated RNA was incubated with the indicated proteins. Experiments were performed 3 times with similar results. h). Temporal expression profiling of *lncPsbO* (left) and *GhPsbO* (right) in WT and *OE-Vd10375N* roots post-*V. dahliae* inoculation (0, 6, 12, and 24 hpi) via qRT-PCR. Data represent means ± SE from 3 replicates. i to j). *lncPsbO* negatively regulates GhPsbO abundance in *N. benthamiana*. Transiently co-expression of GhPsbO-GFP with *35S: lncPsbO* significantly reduced GFP fluorescence compared to empty (*35S)* control, confocal microscopy was conducted 48 hours postinfiltration. Scale bar, 30 µm (i). Protein stability as shown by western blotting (j). k) Quantitative RT-PCR analysis of the expression levels of *lncPsbO* and GhPsbO in the overexpression lines (Student's *t*-test, ***P < 0.001; ****P < 0.0001). Each data point represents the mean value of 3 independent biological replicates per treatment (mean ± SE), error bars represent ± SE of the dataset.

Plants respond to pathogen invasion by activating defense-related pathways, including the production of phytohormones and defense proteins (Schulze et al. 2022; Huang et al. 2024). Given that GhPsbO physically interacts with Vd10375 and forms a protein complex, we examined whether Vd10375 influences *GhPsbO* expression to trigger host immunity. Co-expression assays in *N. benthamiana* revealed that GhPsbO-Flag protein levels were markedly elevated when co-expressed with Vd10375N-YFP-HA, compared to the YFP-HA control (Fig. 4a). This result indicates that GhPsbO interacts with Vd10375N and enhances its stability.

To further investigate this stabilization effect, we performed in vitro degradation assays using total protein extracts from *OE-Vd10375N* and WT cotton plants. Recombinant GhPsbO-His protein displayed significantly reduced degradation in *OE-Vd10375N* extracts relative to WT controls (Fig. 4b). Western blot analysis confirmed consistently higher GhPsbO levels in 3 independent OE-Vd10375N lines compared to WT (Fig. 4c), indicating that Vd10375N enhances GhPsbO stability.

Previous studies have shown that TaWKS1-mediated phosphorylation of PsbO at conserved residues (T104 and T245) leads to its degradation via cysteine and aspartic proteases (Wang et al. 2019). We hypothesized that Vd10375N interfered with this degradation pathway. AlphaFold-predicted docking analysis revealed that the Vd10375N-GhPsbO interaction interface overlapped with T109—homologous to wheat PsbO's T104 site—suggesting that Vd10375N masks critical degradation-prone residues (Figure S5). We also assessed whether Vd10375 influences GhPsbO expression at the transcriptional level. *GhPsbO* transcript levels were significantly higher in cotton roots infected with wild-type *V. dahliae* Vd991 than those infected with the *ΔVd10375* mutant (Fig. 4d), supporting a role for Vd10375 in transcriptional upregulation of *GhPsbO*.

Given Vd10375's RNase activity, we speculated that it may stabilize GhPsbO by degrading negative regulators. LncRNA-seq analysis of *OE-Vd10375N* versus WT plants identified differentially expressed long noncoding RNAs (lncRNAs), with a focus on those significantly downregulated. Gene Ontology and KEGG pathway analyses showed that the target genes of these differentially expressed lncRNAs were enriched in photosynthesis-related pathways (Figure S6). Among them, the antisense transcript of GhPsbO is annotated as a long noncoding RNA (*lncPsbO*) with a full-length of 1,048 nucleotides, which is significantly suppressed in OE-Vd10375N plants (Fig. 4e). *LncPsbO* exhibits 76% complementarity to the full-length sequence of GhPsbO mRNA, and is characterized as a single-exon long noncoding RNA containing no introns. Quantitative RT-PCR (qRT-PCR) analysis showed that the basal expression level of lncPsbO in uninfected cotton plants was approximately 20% of the GhPsbO mRNA abundance (Figure S7a). RNA immunoprecipitation followed by qPCR (RIP-qPCR) demonstrated strong in vivo binding between Vd10375N and *lncPsbO*, with significant *lncPsbO* enrichment in Vd10375N pull-downs (Fig. 4f). To determine whether Vd10375N interacts with GhPsbO or lncPsbO transcripts, we performed in vitro RNA binding assays using GST-tagged recombinant Vd10375N protein together with biotin-labeled GhPsbO mRNA or lncPsbO RNA. Due to the strong nuclease activity of Vd10375N, multiple attempts were unsuccessful. Therefore, we substituted the Vd10375Nm protein for validation, which showed that Vd10375Nm associates with lncPsbO but not GhPsbO mRNA in vitro (Fig. 4g).

qRT-PCR confirmed that *OE-Vd10375N* plants exhibited lower *lncPsbO* but higher *GhPsbO* transcript levels compared to WT (Fig. 4h), and *lncPsbO* transcript levels were significantly lower in cotton roots infected with wild-type Vd991 than those infected with the *ΔVd10375* mutant (Figure S7b), suggesting that Vd10375N enhances *GhPsbO* expression by repressing *lncPsbO*. The double-stranded RNA immunoprecipitation experiment demonstrated that *GhPsbO* and *lncPsbO* bind to form double-stranded RNA in cotton (Figure S7c).

Previous studies have shown that pathogen-derived noncoding RNA suppress host immunity by degrading host RNAs (Zhang et al. 2016; Cai et al. 2018). To validate the inhibitory effect of *lncPsbO* on *GhPsbO*, we transiently co-expressed *GhPsbO*-GFP with *lncPsbO* in *N. benthamiana*, which resulted in reduced GFP fluorescence and GhPsbO protein levels (Fig. 4, i and j). Moreover, *GhPsbO* transcript levels were lower in OE-*lncPsbO* roots than in WT (Fig. 4k), indicating that *lncPsbO* acts in *trans* to suppress *GhPsbO*. Collectively, these findings establish that Vd10375 inhibits GhPsbO degradation via post-transcriptional and transcriptional regulation.

### *GhPsbO* knockout enhances resistance to *V. dahliae* via ROS accumulation

**Figure. 5.**
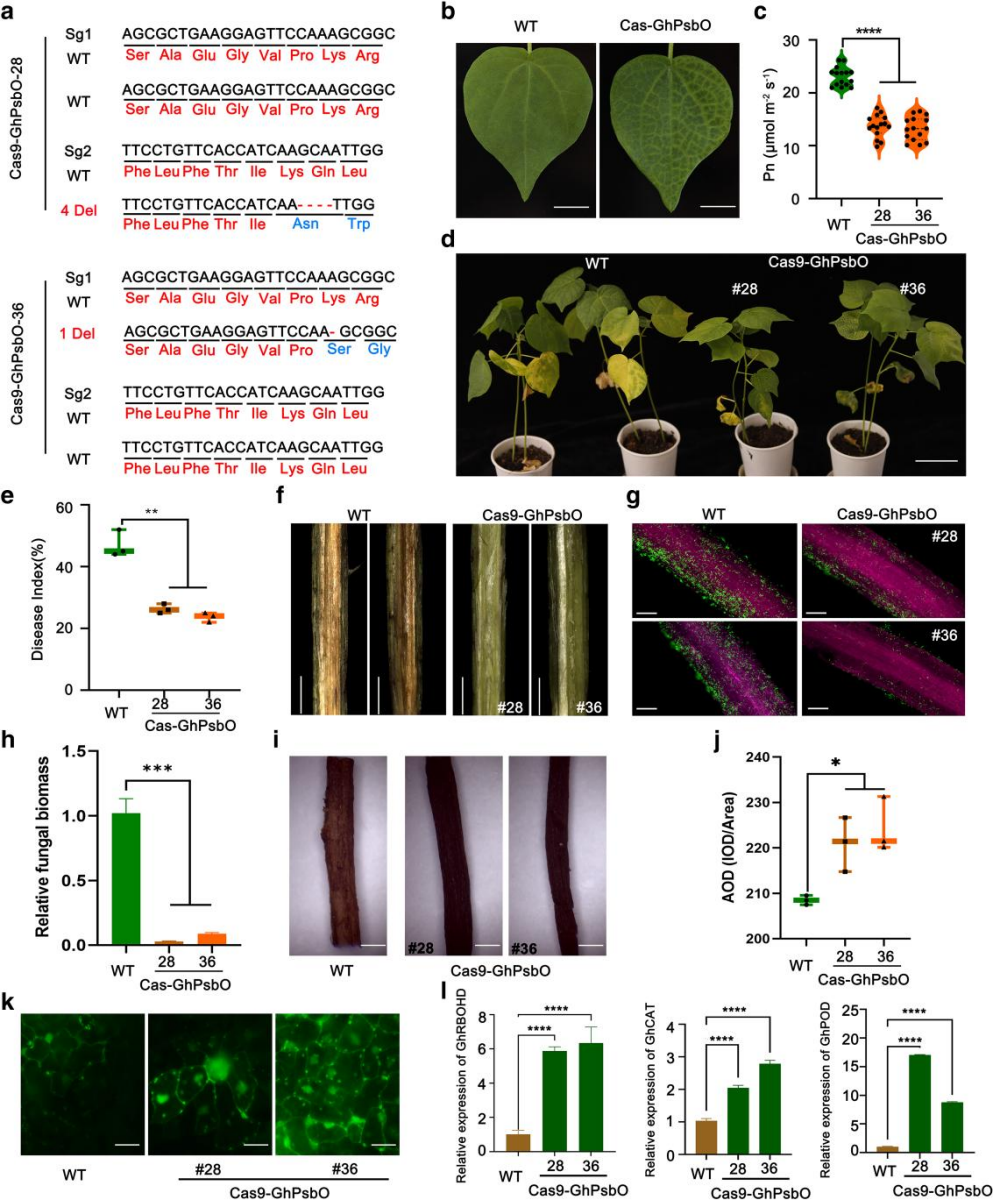
Knockout *GhPsbO* enhances resistance to *V. dahliae* but reduces photosynthetic efficiency. a) CRISPR/Cas9-mediated editing of *GhPsbO* generated 2 independent knockout lines, *Cas9-GhPsbO-28* and *Cas9-GhPsbO-36*, with nucleotide deletions at the targeted loci (indicated by “−”). b) Phenotypic analysis showed whitening along leaf veins in *Cas9-GhPsbO* plants, suggesting impaired chloroplast function. Scale bar = 1 cm. c) Field-based measurements demonstrated that the net photosynthetic rate (Pn) was significantly reduced in *Cas9-GhPsbO* plants compared to WT (Student's *t*-test, ****P < 0.0001). Each data point represents an averaged value from 3 biological replicates with error bars indicating standard error. (*n* = 15). d). Disease symptoms of leaves of WT and *GhPsbO*-edited cotton plants at 24 d postinoculation (dpi). Scale bar = 5 cm. e) Disease index of infected cotton plants edited with *Cas9-GhPsbO,* along with the WT, at 24 dpi. f) Longitudinal stem sections of infected plants at 24 dpi indicated less vascular browning in *Cas9-GhPsbO* lines, consistent with lower pathogen colonization. Scale bar = 2 mm. g). Confocal microscopy confirmed reduced *V. dahliae* Vd991-GFP biomass in roots of *Cas9-GhPsbO* plants at 24 dpi. Scale bar = 100 μm. h) Quantitative PCR analysis of relative biomass following *V. dahliae* infection. Infected roots were collected at 24 dpi, after which DNA was isolated and qPCR analysis was performed (Student's *t*-test, ***P < 0.001). Each data point represents the mean value of 3 independent biological replicates per treatment (mean ± SE), error bars represent ± SE of the dataset. i) Root DAB staining revealed increased reactive oxygen species (ROS) accumulation in *Cas9-GhPsbO* compared to WT and *OE-GhPsbO* plants. j) Images obtained after DAB staining were quantified using ImageJ software. Scale bar = 2 mm. (Student's *t*-test, *P < 0.05). Each data point represents the mean value of 3 independent biological replicates per treatment (mean ± SE), error bars represent ± SE of the dataset. k) ROS burst assays using H_2_DCF-DA staining following flg22 treatment showed stronger ROS signals in *Cas9-GhPsbO* leaves, indicating enhanced immune responsiveness. Scale bar = 50 μm. l) Expression levels of defense-associated ROS-regulating genes—*GhRBOHD*, *GhPOD*, and *GhCAT*—were elevated in *Cas9-GhPsbO* roots upon *V. dahliae* infection (Student's *t*-test, ****P < 0.0001). Each data point represents the mean value of 3 independent biological replicates per treatment (mean ± SE), error bars represent ± SE of the dataset.

To dissect GhPsbO's role in defense, we generated CRISPR-Cas9 knockout lines (*Cas9-GhPsbO-28* and *Cas9-GhPsbO-36*), with mutations verified by Sanger sequencing (Fig. 5a). *Cas9-GhPsbO* plants exhibited visible vein-centered leaf chlorosis, reduced photosynthetic efficiency, and compromised root development (Fig. 5, b and c and Figure S8a). Surprisingly, these plants displayed increased resistance to Vd991, as evidenced by reduced disease symptoms and vascular browning (Fig. 5, d—to f), and lower fungal biomass in roots (Fig. 5, g and h).

Given that ROS accumulation is a known defense mechanism and previous work implicates PsbO deficiency in elevated ROS (Wang et al. 2019), we examined ROS levels in the mutants. DAB staining revealed increased ROS accumulation in both roots and leaves of infected mutants (Fig. 5i and Figure S8b). The images were converted to grayscale, and the intensity of DAB staining was quantified using ImageJ software (Fig. 5j and Figure S8c). Flg22-induced ROS bursts were significantly stronger in Cas9-GhPsbO plants than in WT, as shown by DCF staining (Fig. 5k). Furthermore, ROS-related genes were upregulated in *Cas9-GhPsbO* plants during infection (Fig. 5l). These results suggest that *GhPsbO* disruption impairs photosynthesis and development, but simultaneously fosters a high-ROS environment unfavorable to *V. dahliae*, thereby enhancing resistance.

### GhMYB44 promotes resistance by repressing *GhPsbO* expression

**Figure. 6.**
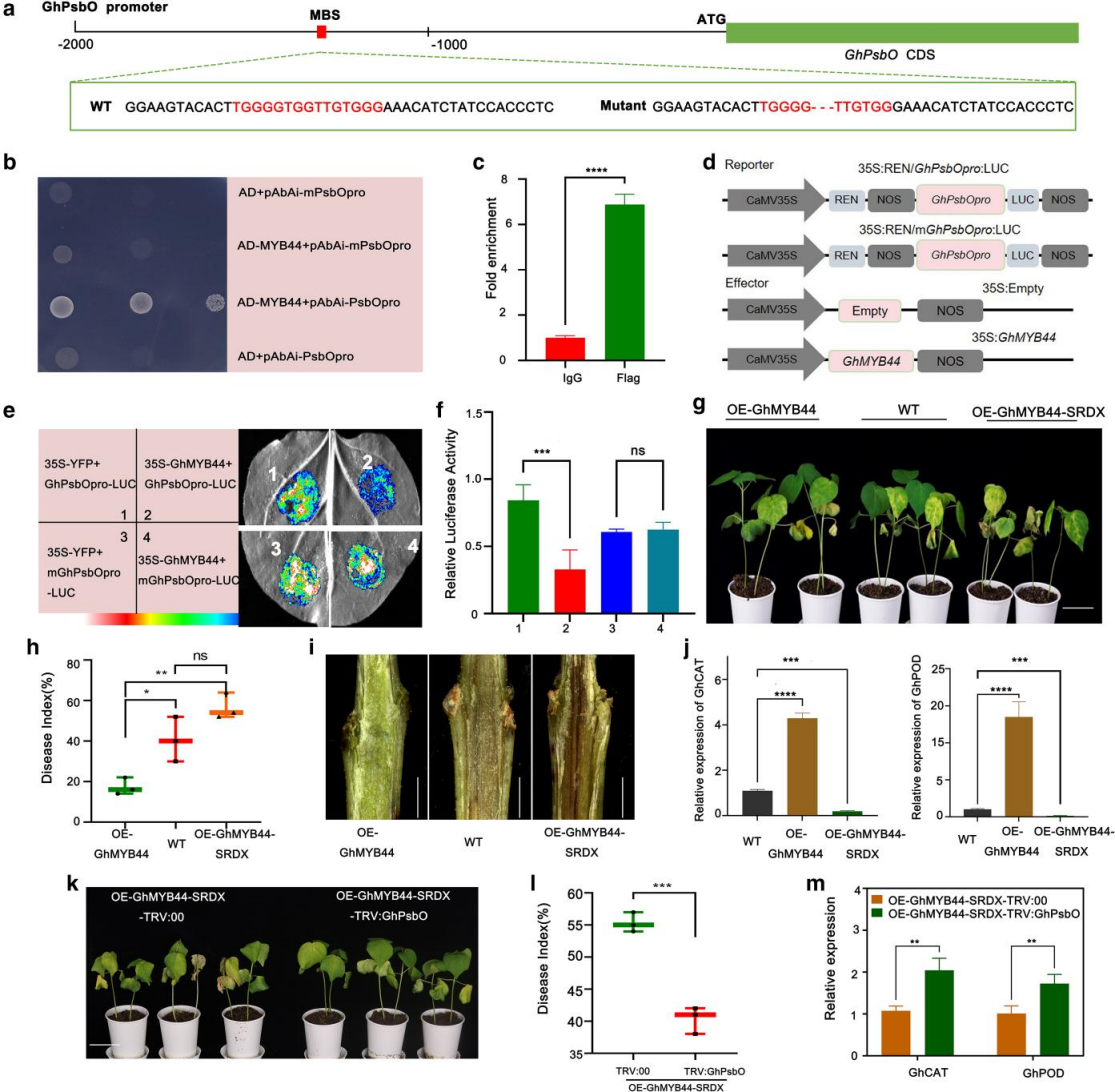
GhMYB44 binds to the *GhPsbO* promoter and represses its expression and enhances disease resistance. a) Schematic illustration of the *GhPsbO* promoter showing the MYB-binding cis-element recognized by GhMYB44. b) Yeast 1-hybrid (Y1H) assay demonstrated direct binding of GhMYB44 to the *GhPsbO* promoter. Y1HGold yeast co-transformed with *GhPsbOpro*–pAbAi and AD-GhMYB44 grew on selective medium containing 400 ng mL^−1^ AbA. c). Chromatin immunoprecipitation followed by qPCR (ChIP-qPCR) in *OE-GhMYB44* cotton confirmed in vivo binding of GhMYB44 to the *GhPsbO* promoter. Anti-HA antibody was used for immunoprecipitation (Student's *t*-test, ****P < 0.0001). Each data point represents the mean of 3 independent experiments in which each experiment contained 3 replicates. The error bars represent s.e.m. (*n* = 3). d to e). Dual-luciferase assays in *N. benthamiana* leaves showed that GhMYB44 represses *GhPsbO* promoter activity *in planta* (Student's *t*-test, ***P < 0.001, ns indicates no significant difference), “35S” represents empty vector, 35S-YFP as the negative control. (f) Quantitative analysis of luminescence intensity in (e). g). Disease symptoms of WT, *OE-GhMYB44*, and *OE-GhMYB44-SRDX* cotton plants at 20 d postinfection (dpi) with *V. dahliae*. Scale bar = 5 cm. h) Disease index in inoculated cotton plants overexpressing *GhMYB44*, specifically silencing cotton plants, and WT following inoculation with *V. dahliae* (Student's *t*-test, * *P* < 0.05, **P < 0.01, ns indicates no significant difference). i) Longitudinal stem sections at 20 dpi confirmed that OE-GhMYB44-SRDX lines displayed greater vascular discoloration. Scale bar = 2 mm. j) Expression of antioxidant genes *GhCAT* and *GhPOD* was significantly elevated in *OE-GhMYB44* lines and suppressed in *OE-GhMYB44-SRDX* plants following infection. (Student's *t*-test, ***P < 0.001; ****P < 0.0001). k to m). Silencing GhPsbO in the OE-GhMYB44-SRDX background using TRV:GhPsbO partially restored resistance, as shown by reduced disease symptoms (k) Scale bar = 5 cm, lower DI (l) (Student's *t*-test, ***P < 0.001), and increased GhCAT and GhPOD expression (m) (Student's *t*-test, **P < 0.01). Each data point represents the mean of 3 independent experiments in which each experiment contained 3 replicates. The error bars represent s.e.m. (*n* = 3).

To uncover transcriptional regulators of *GhPsbO*, we conducted a yeast 1-hybrid (Y1H) screen using the *GhPsbO* promoter as bait. Ten candidate transcription factors were identified (Table S3), among which GhMYB44 emerged as a strong candidate based on its MYB domain and pathogen-responsiveness. RT-qPCR showed that *GhMYB44* expression was rapidly induced in roots following *V. dahliae* infection (Figure S9a). Sequence analysis revealed a MYB-binding motif (TGGGGTGGTTGTGG) in the 2 kb *GhPsbO* promoter region (Fig. 6a).

Y1H assays confirmed GhMYB44 binding to the *GhPsbO* promoter, which was abolished upon mutating the recognition site (Fig. 6b). ChIP-qPCR further validated this interaction *in planta* (Fig. 6c). Dual-luciferase reporter assays demonstrated that GhMYB44 significantly repressed *GhPsbO* promoter activity, an effect lost in the mutated promoter construct (Fig. 6, d—to f).

To assess in vivo regulatory effects, we generated *OE-GhMYB44* and *OE-GhMYB44-SRDX* lines (a dominant-negative repressor) (Figure S9b). Postinfection transcript analysis revealed that *GhPsbO* was significantly differentially expressed in *OE-GhMYB44* and *OE-GhMYB44-SRDX* lines compared to WT (Figure S9c). Functionally, *OE-GhMYB44* plants exhibited significantly reduced disease symptoms, while *OE-GhMYB44-SRDX* lines showed slightly more severe DI than the WT upon Vd991 infection (Fig. 6, g and h). Consistent stem section analysis revealed lighter lesions in *OE-GhMYB44* and darker ones in *OE-GhMYB44-SRDX* lines (Fig. 6i). Moreover, ROS-scavenging genes were upregulated in *OE-GhMYB44* and downregulated in *OE-GhMYB44-SRDX* (Fig. 6j).

To establish genetic epistasis, we silenced *GhPsbO* in the *OE-GhMYB44-SRDX* background, generating *OE-GhMYB44-SRDX*  *×*  *TRV:GhPsbO* plants. These plants displayed attenuated disease symptoms and partially restored expression of ROS-scavenging genes compared to *OE-GhMYB44-SRDX*  *×*  *TRV:00* controls (Fig. 6, k to m). These results confirm that *GhPsbO* functions downstream of GhMYB44 in modulating resistance via ROS regulation. In summary, GhMYB44 enhances disease resistance in cotton by repressing *GhPsbO*, thereby promoting ROS accumulation and defense activation.

## Discussion

**Figure. 7.**
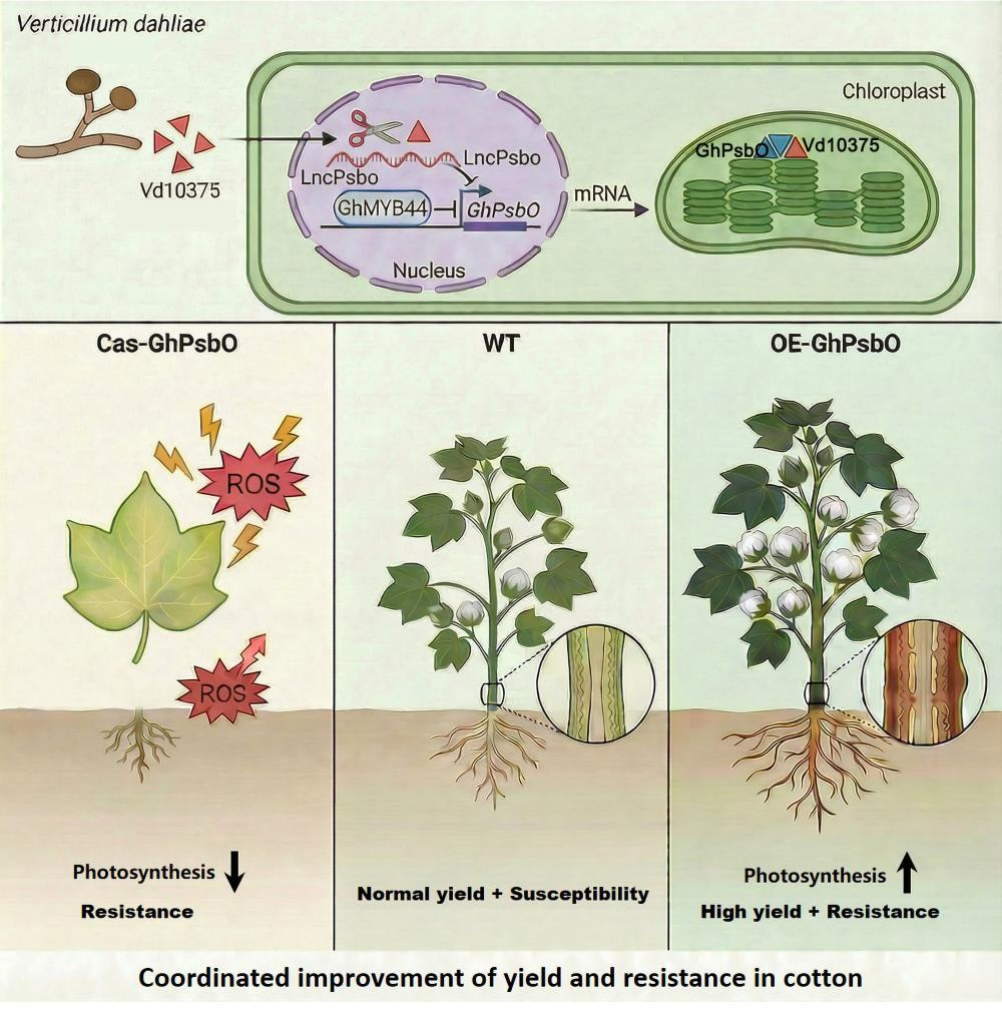
Proposed model illustrating how GhPsbO breaks the growth–immunity tradeoff by simultaneously enhancing yield and defense. During *V. dahliae* infection, mutation of *GhPsbO* compromises photosynthesis and induces excessive superoxide accumulation, thereby elevating ROS levels which reinforce defense responses at the expense of growth. The fungal effector Vd10375 stabilizes GhPsbO by inhibiting its antisense long noncoding RNA (*lncPsbO*) while preventing the degradation of the GhPsbO protein. This effectively suppresses the ROS burst and immune activation triggered by GhPsbO degradation, allowing the pathogen to evade the innate immune response of cotton. Conversely, excessive accumulation of GhPsbO significantly boosts plant photosynthetic efficiency, accelerates lignin biosynthesis and deposition in cell walls, and enhances plant structural defense, ultimately manifesting as both improved yield and enhanced disease resistance.

In agricultural systems, crop yield and disease resistance often exhibit an antagonistic relationship: high-yielding varieties tend to be more susceptible to disease, while disease-resistant cultivars commonly suffer from reduced productivity (He et al. 2022; Li et al. 2022). Overcoming this inherent tradeoff remains a central challenge in modern crop breeding. While some resistance-related genes have been shown to improve immunity without compromising yield, genes that simultaneously enhance both yield and disease resistance remain rare. Here, we uncover a key regulatory mechanism mediated by the chloroplast protein GhPsbO, which serves as a dual-function integrator of these 2 essential traits. Overexpression of the GhPsbO protein, driven by a constitutive promoter, significantly enhances photosynthetic efficiency in plants, accelerates the biosynthesis and deposition of lignin in cell walls, and strengthens the plant's structural defense capabilities, ultimately resulting in increased yield and enhanced disease resistance. Our findings reveal an actionable regulatory network centered on GhPsbO, offering a blueprint for breeding elite cultivars with concurrent improvements in both productivity and immunity. The mechanism by which GhPsbO balances cotton growth and immunity, as proposed in this study, is shown in Fig. 7.

### GhPsbO is a pleiotropic regulator that coordinates disease resistance and yield

The interaction between *V. dahliae* and its host reflects a dynamic molecular arms race, characterized by a continuous relationship between pathogen-secreted effectors and host defense systems (Klosterman et al. 2011; Liu et al. 2024) . However, the intracellular functions of many fungal effectors remain poorly defined. Through secretome analysis of the *V. dahliae* strain Vd991, we identified Vd10375 as a key virulence factor. The presence of Vd10375 homologs in diverse fungal genera, including *Fusarium* and *Clonostachys* (Figure S2a), suggests that this effector family is evolutionarily conserved and functionally significant during infection. Yeast 2-hybrid screening identified GhPsbO, a chloroplast-localized protein, as a specific interactor of Vd10375. Functional validation revealed that *GhPsbO* overexpression simultaneously enhances resistance to *V. dahliae.* This resistant phenotype illustrates an endogenous “invasion–defense” mechanism in plant–pathogen interactions, whereby the host protein GhPsbO recognizes the fungal effector Vd10375 to counteract infection. A conceptually similar defense strategy was recently reported, in which the host protein OsCPIP8 recognizes the effector XoCysp1 upon infection, leading to its activation and subsequent enhancement of immunity (Zhang et al. 2025). Notably, overexpression of GhPsbO also improves agronomic traits, such as plant height, stem thickness, branching, and boll number. These findings establish GhPsbO as a previously uncharacterized pleiotropic regulator that synergizes immunity and yield, underscoring its value for molecular breeding aimed at dual-trait improvement.

GhPsbO is known to be targeted for degradation through phosphorylation-mediated proteolysis under stress (Wang et al. 2019). To explore whether its stability is functionally relevant to immunity, we investigated its interaction with Vd10375. Our data reveal that Vd10375 exerts a dual-layered regulatory mechanism: it both inhibits GhPsbO protein degradation and facilitates degradation of *lncPsbO*, a negative post-transcriptional regulator of GhPsbO. It is worth noting that complex and diverse regulatory mechanisms exist between lncRNAs and their cognate transcripts (Jin et al. 2021; Lucero et al. 2021; Kiger and Schroeder 2024). For example, SVALKA finely regulates the transcriptional response to low temperature through 3 mechanisms: transcriptional collision and dsRNA- and DICER-mediated pathways that regulate the CBF1 transcription factor, and interaction with Polycomb Repressor Complex 2 to control histone methylation of CBF3 (Kindgren et al. 2018; Zacharaki et al. 2023; Kiger and Schroeder 2024). Our study demonstrates that *lncPsbO* suppresses *GhPsbO* transcription by forming double-stranded RNA with its coding strand (Figure S7c). Similar to SVALKA, lncPsbO is transcribed antisense and partially overlaps with GhPsbO, a genomic arrangement that permits *cis*-regulation and possible Pol II collision, which will be experimentally characterized in our future work.

Interestingly, *GhPsbO* knockout mutants also displayed enhanced resistance, comparable to overexpression lines. This apparent paradox can be explained by GhPsbO's essential role in Photosystem II (PSII), where it contributes to the oxygen-evolving complex (Popelkova and Yocum 2011). Loss of *GhPsbO* likely destabilizes PSII, leading to excessive reactive oxygen species (ROS) production (Krieger-Liszkay et al. 2008), which in turn enhances immunity. Therefore, *GhPsbO* overexpression and knockout confer resistance via distinct mechanisms: overexpression promotes immunity by reinforcing photosynthesis and lignification, whereas knockout induces ROS-mediated defense at the cost of yield. While the yield penalty associated with GhPsbO knockout limits its direct breeding utility, the disease resistance and high-yield traits conferred by GhPsbO overexpression offer a promising avenue for future convergent crop breeding.

It should be noted that although traditional views hold that overexpression and loss-of-function mutant phenotypes are opposite, recent studies increasingly indicate that they are not necessarily mutually exclusive. For instance, both loss-of-function and gain-of-function alleles of the soybean *GmIF6H1* gene increase susceptibility to *Phytophthora sojae* by precisely regulating metabolites (Yang et al. 2025). Additionally, both overexpression and knockout of the maize thiamine pyrophosphate kinase ZmTPK2 impair yield and grain quality, indicating that optimal TPP levels are crucial for productivity (Luo et al. 2025). This parallels our observations that both *OE-GhPsbO* and *Cas9-GhPsbO* plants exhibit enhanced disease resistance, suggesting that such overlapping phenotypes may indeed reflect a broader biological phenomenon.

Given GhPsbO's predominant expression in roots, its role in root-specific lignin biosynthesis warrants further investigation. Supporting our findings, studies in rice and wheat have confirmed the defensive function of ROS bursts (Qi et al. 2019; Xiao et al. 2023). For example, the rice resistance gene *Pijx* confers broad-spectrum protection against panicle blast through OsRbohC-mediated ROS generation (Xiao et al. 2023). These findings underscore the dual nature of ROS as both a defense signal and a potential source of cellular damage, highlighting the importance of fine-tuning ROS homeostasis in future disease resistance breeding efforts.

### Chloroplasts as regulatory hubs integrating immunity and yield

While the role of nuclear-localized transcription factors in plant immunity is well established, emerging evidence places chloroplasts at the center of growth-defense coordination (Kachroo et al. 2021). Pathogen effectors frequently target chloroplast-localized proteins to manipulate host responses. For instance, the coat protein of PMMoV targets the chloroplast outer membrane protein OMP24 in pepper, triggering ROS production and conferring resistance (Han et al. 2023). Given the chloroplast's dual role as a photosynthetic engine and immune modulator, we hypothesized that chloroplast proteins offer unique opportunities for simultaneous enhancement of yield and disease resistance.

Supporting this view, recent work on potatoes has shown that RNAi-mediated silencing of chloroplast elongation factors *StTuA* and *StTuB* increased susceptibility to *Phytophthora infestans*, impaired photosynthesis, and reduced tuber yield (Qi et al. 2024). Conversely, overexpression of these genes enhanced chloroplast translation, stimulated photosynthesis, and promoted both ROS production and immune responses—thus improving resistance and productivity in parallel. In a mechanistically similar manner, our study demonstrates that GhPsbO promotes both yield and immunity via enhancing photosynthesis and lignin synthesis. Collectively, these results position chloroplast-resident proteins as high-value targets for trait pyramiding and holistic crop improvement strategies.

Beyond chloroplasts, recent findings also implicate mitochondrial proteins in disease resistance (Yang et al. 2022; Xiao et al. 2023). For example, the rice resistance gene *Pijx* functions through 26S proteasome-mediated degradation of mitochondrial ATP synthase subunit beta, triggering OsRbohC-dependent ROS bursts and conferring broad-spectrum resistance (Xiao et al. 2023). Similarly, the tomato mitochondrial PPR protein RTP7 enhances resistance by regulating mitochondrial ROS through alternative splicing of *nad7* transcripts (Yang et al. 2022). These organellar mechanisms collectively highlight the potential of targeting chloroplasts and mitochondria to overcome the tradeoff between growth and defense.

Given the central roles of organellar proteins in coordinating immunity and growth, future breeding efforts should leverage multigene pyramiding, CRISPR-based multiplex genome editing, and synthetic biology to engineer these targets combinatorially. For example, saturated base editing of *PsbO*, *StTuA*, and *StTuB* could help identify elite alleles with superior regulatory functions and enhanced breeding potential.

In conclusion, this study identifies GhPsbO as a pivotal integrator of disease resistance and yield in cotton. We elucidate a previously uncharacterized mechanism whereby this chloroplast-localized protein orchestrates immune defense while sustaining photosynthetic productivity. By simultaneously breaking the long-standing tradeoff between growth and defense, GhPsbO provides a promising molecular target and a strategic framework for next-generation crop improvement.

## Materials and methods

### Fungal strains and plant materials

The *Verticillium dahliae* strain Vd991 (Liu et al. 2021) was used throughout this study. *V. dahliae* was cultured on potato dextrose agar at 25 °C in the dark. For conidia collection, the fungus was grown in Czapek Dox liquid medium at 25 °C with shaking at 180 rpm for 5 to 7 d. Upland cotton (*Gossypium hirsutum*) cultivars “JM11,” “ZM24”were grown in a greenhouse under 16 hours of light/8 hours of dark cycles at 26 °C. *Nicotiana benthamiana* plants were cultivated under identical photoperiods at 22 ± 1 °C (Song et al. 2021). Four-wk-old cotton plants were inoculated with a 1 × 10^7^ conidia ml^−1^ conidial suspension using a root-dip method (Liu et al. 2021). The DI was calculated following the previously described protocols (Ma et al. 2025). Specifically, the DI for cotton plants was calculated using the following formula: DI (%) = [(∑disease grades × number of infected plants)/(total number of checked plants × 4)] × 100. Cotton seedlings were classiﬁed into 5 severity levels (grades 0, 1, 2, 3, and 4) according to the symptom severity observed on cotyledons and true leaves.

### Yeast signal sequence trap assay

To validate the signal peptide (SP) functionality of Vd10375, a yeast invertase secretion assay was employed (Nie et al. 2019). The SP sequence of Vd10375 was cloned into the pSUC2 vector using specific primers (Table S3), and the resulting construct, pSUC2-Vd10375^SP^, was transformed into the yeast strain YTK12. Transformants were first selected on CMD/-W medium (0.67% yeast nitrogen base without amino acids, 0.075% W dropout supplement, 2% sucrose, 0.1% glucose, 2% agar), then transferred to YPRAA medium (2% raffinose, 2% peptone, 1% yeast extract, 2 mg mL^−1^ antimycin) for invertase secretion assessment. pSUC2-Avr1b^SP^ and empty pSUC2 vectors were used as positive and negative controls, respectively. Invertase activity was further confirmed by TTC (2,3,5-triphenyltetrazolium chloride) reduction.

### Cotton transformation

Cotton transformation experiments were performed at the transgenic platform of the State Key Laboratory of Cotton Biology, Chinese Academy of Agricultural Sciences (Ge et al. 2023). The constructs pCAMBIA2300-Vd10375N-Flag, pCAMBIA2300-GhMYB44-Flag, pCAMBIA2300-GhMYB44-SRDX, WMV068-GhPsbO-HA (OE-GhPsbO), and WMC016-GhPsbO (Cas9-GhPsbO) were introduced into *G. hirsutum* cultivar ZM24 via Agrobacterium-mediated transformation (Agrobacterium tumefaciens strain EHA105) (Ge et al. 2023). Transgenic lines carrying homozygous single-copy insertions were identified by PCR. Two or 3 independent transgenic lines were subsequently used for *V. dahliae* inoculation.

### Transient expression in *N. benthamiana*

Recombinant constructs were introduced into *Agrobacterium tumefaciens* strain GV3101. Agrobacterium strains containing the indicated constructs were cultured at 28 °C at 220 rpm for 10 h, and then were collected by centrifugation at 23 °C and 5,000 × *g* for 5 min. Agrobacterium cells were adjusted to an OD_600_ of 0.5 in MMA buffer (10 mM MgCl_2_, 100 μM acetosyringone, 10 mM MES, pH = 5.6) and allowed to stand in the dark for 2 h before plant transformation. Five-wk-old *N. benthamiana* leaves were infiltrated for transient expression.

### Measurement of ROS accumulation

ROS burst assays were performed as described previously (Wang et al. 2019). Leaves from 4-wk-old *OE-Vd10375N* transgenic and wild-type plants were incubated in distilled water overnight. ROS production was induced with 100 nM flg22 and monitored using a luminol-based chemiluminescence assay. Luminescence was measured at 10-second intervals for 20 min using a Glomax 20/20 luminometer (Promega), with 3 biological replicates per treatment.

### Subcellular localization

Coding sequences of *Vd10375N* and *GhPsbO* were amplified from cDNA and fused to fluorescent tags under the control of the *CaMV 35S* promoter. Constructs were introduced into GV3101 and infiltrated into *N. benthamiana* leaves. After 48 h in darkness, fluorescence was visualized using an LSM780 confocal microscope.

### GUS staining assay

GUS staining was conducted as described previously (Zhang et al. 2024a). Cotton roots were fixed in 90% (v/v) precooled acetone for 20 min, rinsed 3 times, and incubated overnight in staining buffer containing 1 mM X-Gluc at 37 °C. Samples were cleared with 95% ethanol and imaged using a digital camera.

### RT-qPCR analysis

Total RNA was extracted from cotton roots using the RNAprep Pure Plant kit (Tiangen, Beijing, China) and reverse-transcribed using HiScript III All-in-One RT SuperMix (Vazyme, Nanjing, China). RT-qPCR was conducted with ChamQ Universal SYBR qPCR Master Mix (Vazyme). Primers were designed using a previously established primer database. *GhUBQ* (*Gh_D11G1120*) served as the internal reference gene, and relative expression was calculated using the 2^−ΔΔct^ method (Rieu and Powers 2009).

### Generation of deletion and complementation strains

To construct the *ΔVd10375* mutant, upstream and downstream sequences of *Vd10375* were amplified and fused with a hygromycin resistance cassette using SOE-PCR. The final fragment (*Vd10375-UP*/*Hyg*/*DOWN*) was cloned into the B303 vector (Wen et al. 2023) and transformed into *Agrobacterium tumefaciens* AGL-1 for ATMT (Wang et al. 2016). For complementation, the full *Vd10375* genomic region, including 1.5 kb upstream, was cloned into the pCHG vector and reintroduced into *ΔVd10375* via ATMT (Zhou et al. 2017).

### Yeast 2-hybrid assay

Y2H assays were performed using the GAL4 system (Yang et al. 2020). The coding region of *Vd10375* (excluding its SP) and *GhPsbO* were cloned into pGBKT7 and pGADT7 vectors, respectively. Construct pairs were co-transformed into AH109 yeast and selected on SD/-Leu/-Trp medium, followed by interaction screening on SD/-Leu/-Trp/-His medium.

### Co-immunoprecipitation assay

The *Agrobacterium* strain containing Vd10375N-YFP-HA or YFP-HA and GhPsbO-Flag constructs were co-infiltrated into *N. benthamiana* leaves in a 1:1 ratio (Zhang et al. 2019). After 48 h, leaves were ground in liquid nitrogen, and total proteins were extracted in IP buffer (1 M Tris-HCl, pH 7.5, 5 M NaCl, 1 M MgCl_2_, 10% Tween-20, 1 M DTT, protease inhibitor cocktail). Samples were incubated with anti-DDDDK-tag magnetic beads for 4 hours at 4 °C, washed, and analyzed by SDS-PAGE followed by immunoblotting with anti-Flag and anti-HA antibodies.

### Protein degradation assay

For in vivo degradation, 5 μg of recombinant GhPsbO-His protein was incubated with total protein extracts from Vd10375N transgenic or ZM24 control plants at 28 °C for 0, 30, 60, and 120 min. Degradation was analyzed by Western blot using anti-His antibodies.

### RNA immunoprecipitation

RIP assays were performed using the RNA-Binding Protein Immunoprecipitation Kit (EMD Millipore, USA) to detect interactions between *lncPsbO* and Vd10375. Incubate leaf lysates from the OE-Vd10375-HA line with anti-HA antibody or control IgG antibody, and then pulled down with protein A agarose beads. RNA was purified and quantified via RT-qPCR.

### Double-stranded RNA immunoprecipitation

Fully grind the cotton leaf tissue before proceeding with lysis. For each immunoprecipitation, the J2 antibody (specific for double-stranded RNA), purchased from Starter, China, with a working dilution of 1:500 for immunoprecipitation, or isotype control IgG was pre-incubated with Protein A/G magnetic beads. The antibody-coated beads were then incubated with cell lysates to capture dsRNA-containing complexes. After extensive washing, bound RNA was digested with Proteinase K and extracted using TRIzol. Purified RNA was analyzed by RT-qPCR for enrichment of specific dsRNA targets, and *GhActin* was used as a negative control.

### Dual-luciferase reporter assays

LUC and REN luciferase activities were measured as previously described (Hellens et al. 2005). A 1,500 bp promoter fragment of GhPsbO was cloned into the pGreen0800-LUC reporter vector. Effector constructs (35S:GhMYB44 or empty vector) were co-infiltrated into *N. benthamiana* leaves alongside the reporter. After 24 h in the dark, followed by 24 h in light conditions, luminescence was imaged using the Tanon 5200 Multi Chemiluminescent Imaging System. Luciferase activity was quantified using the Dual-Luciferase Reporter Assay System (Promega), with firefly LUC normalized to REN. Experiments were repeated 3 times independently.

### Y1H assay

Y1H assays were used to confirm the interaction between GhMYB44 and the *GhPsbO* promoter. Promoter fragments containing either the native or mutated GhMYB44-binding motif were cloned into the pAbAi reporter vector. Full-length *GhMYB44* was cloned into the pGADT7 vector. The bait vectors were introduced into the Y1HGold yeast strain, and transformants were selected on SD/-Ura medium with AbA to suppress self-activation. Interaction was assessed by co-transforming with GhMYB44-pGADT7 and observing growth on SD/-Leu medium with AbA.

### Chromatin immunoprecipitation (ChIP) assay followed by qPCR (ChIP-qPCR)

ChIP assays were performed as previously described (Saleh et al. 2008). Briefly, 2 g of plant tissue was fixed with 1% formaldehyde, lysed, and chromatin was sheared by sonication to 200 to 500 bp fragments. Chromatin was immunoprecipitated with anti-FLAG (DYKDDDDK; Engibody, AT1748) or control rabbit IgG antibodies. Input DNA was reserved for normalization. DNA enrichment was quantified via qPCR using ChamQ SYBR Color qPCR Master Mix, and enrichment was expressed relative to input. Primer sequences are listed in Table S4.

### Phylogenetic analysis

Phylogenetic analysis of Vd10375 was conducted as previously described (Shang et al. 2024). Eighteen homologous protein sequences, including those from *Verticillium*, *Fusarium* and *Clonostachys*, were retrieved from the NCBI database (https://www.ncbi.nlm.nih.gov/). Multiple sequence alignment was performed using ClustalW2. The phylogenetic tree was established by MEGA-11 (https://www.megasoftware.net/) using the maximum likelihood method with 1,000 bootstraps. All sequences of Vd10375 homologs are provided in Table S5, and the alignment used for phylogenetic tree and machine-readable tree file from phylogenetic tree are provided in Files S1 and S2.

### Statistical analysis

All data are shown as means ± standard deviation from at least 3 biological repeats or from 3 technical replicates in 1 of 3 experiments with similar results. Two-tailed Student's *t*-test was used for comparing means between 2 samples. One-way ANOVA (Analysis of variance) was used for testing the significance of the difference among different group means (different lowercase letters indicate significant differences, *P* < 0.05). Detailed statistical reports are provided in Table S6.

### Accession numbers

Sequence data from this article can be found in the CottonMD (A Multiomics Database for cotton biological study) databases under the following accession numbers: GhPsbO (Ghir_D11G019970), GhMYB44 (Ghir_D05G026680). The lncRNA-seq data used in the study were deposited at the National Genomics Data Center database under the accession number CRA069378 and can be downloaded from https://ngdc.cncb.ac.cn/gsa/search?searchTerm=CRA042364. The metabolomics sequencing data has been deposited in MetaboLights under accession number MTBLS14410 and can be downloaded from https://www.ebi.ac.uk/metabolights/MTBLS14410.

## Supplementary Material

koag190_Supplementary_Data

## Data Availability

Data are available in a repository and can be accessed using a unique identifier other than a DOI.
