# Supplementary material for: GhPsbO breaks the growth-immunity tradeoff by simultaneously promoting growth and defense in cotton
Source: Plant Cell. 2026 Jun 19;38(7):koag190. doi: 10.1093/plcell/koag190 (PMC13412046; doi:10.1093/plcell/koag190)
Supplement: koag190_Supplementary_Data [file koag190_supplementary_data.zip › Supplementary Data.pdf]

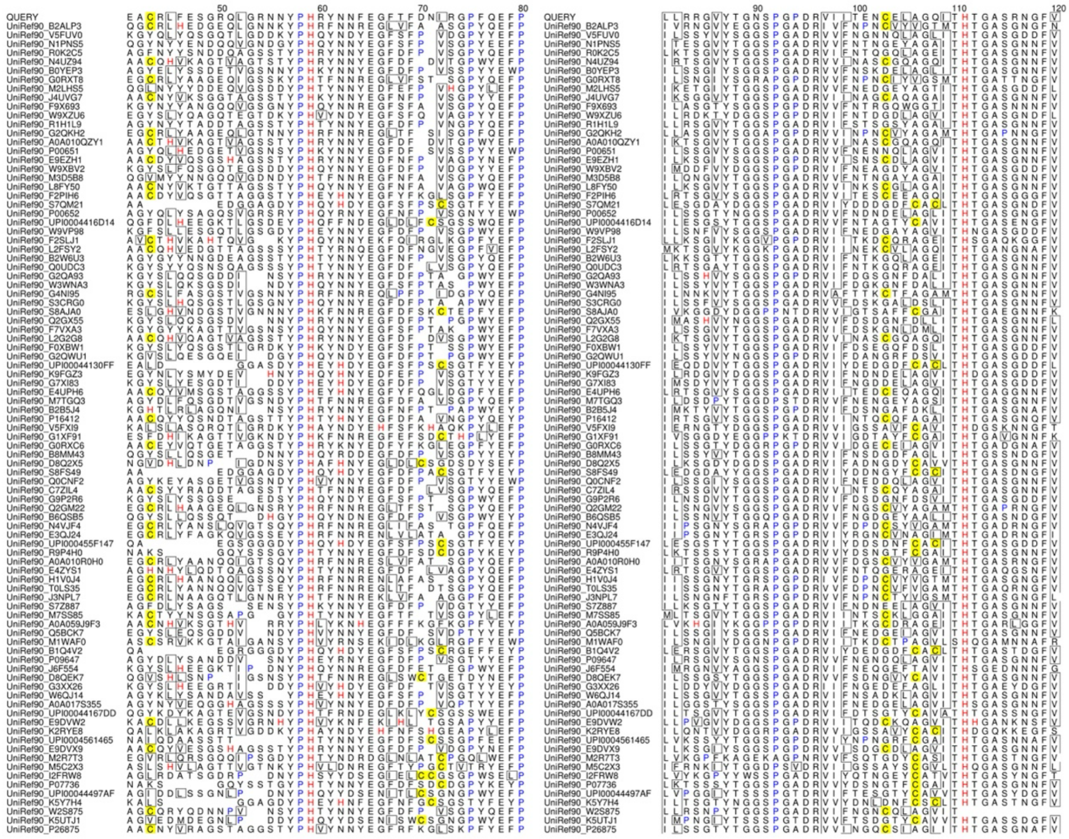

Supplementary Fig. S1. Amino acid conservation analysis of Vd10375 protein. The amino acid sequence of Vd10375 was analyzed using UniProt, revealing five conserved residues within the predicted catalytic domain. These residues are highlighted in blue and red, with their positions labeled above.

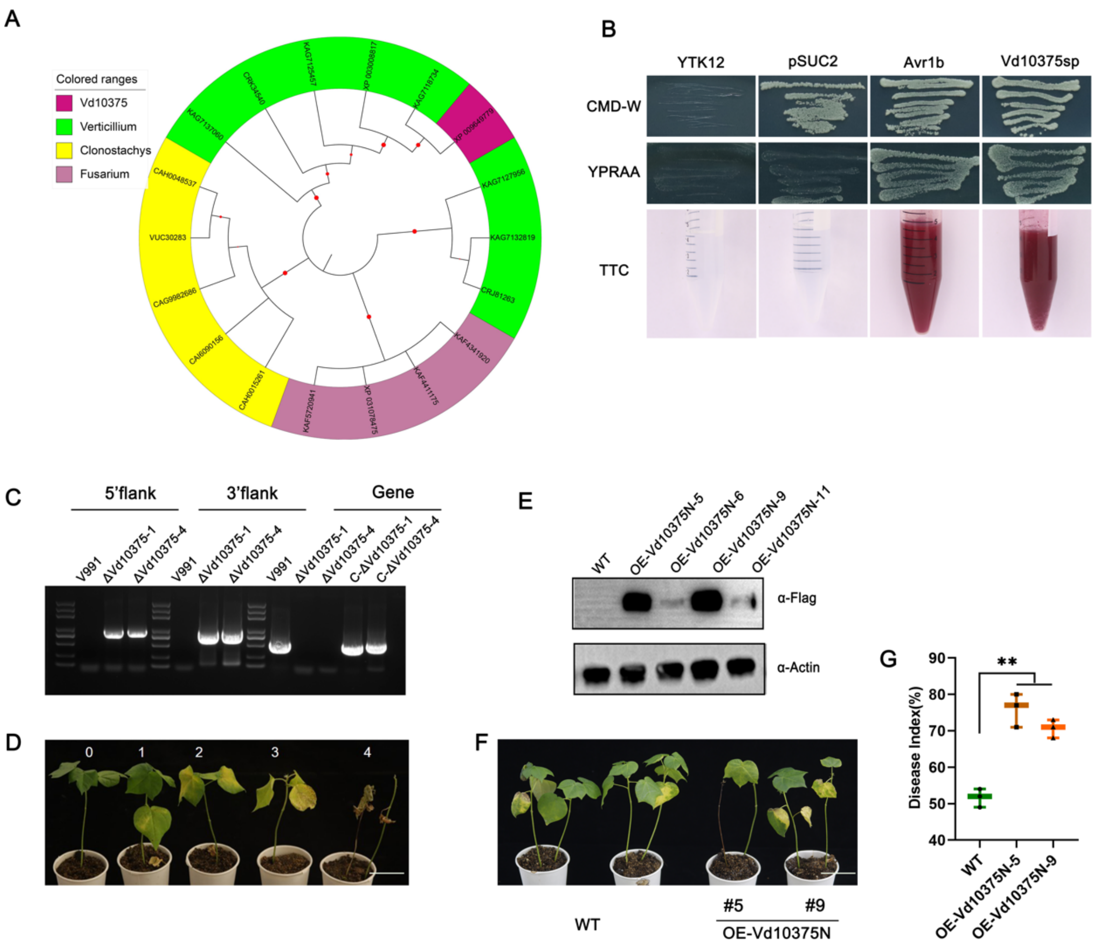

Supplementary Fig. S2. Vd10375 contributes to *V. dahliae* virulence in cotton. A. Phylogenetic tree of Vd10375 and homologs across fungal species. Magenta indicates the target gene Vd10375. **B**. The signal peptide function of Vd10375 (Vd10375SP) was assessed using *Saccharomyces cerevisiae* strain YTK12 transformed with pSuc2t7M13ori vectors carrying *Avr1b* or *Vd10375SP*. Growth on CMD-W (non-selective) and YPRAA (selective) media indicated invertase secretion. Invertase enzymatic activity was further verified by reduction of TTC to red-colored TPF. **C.** PCR verification of *Vd10375* deletion mutants using genomic DNA to confirm 5′/3′ homologous recombination and gene disruption. **D.** Leaf disease symptoms at different rating scales. Scale bar = 5 cm **E.** Western blot detection of *Vd10375* expression in *OE-Vd10375N* transgenic cotton. **F-G**. Disease symptoms and disease index of cotton plants heterologously expressing *Vd10375* following *V. dahliae* inoculation at 28 dpi (Student's *t*-test, $**P < 0.01$), Scale bar = 5 cm, (Error bars

represent ± standard error (SE). Each data point represents the mean disease index from three independent experiments containing 30 plants in total for each treatment.

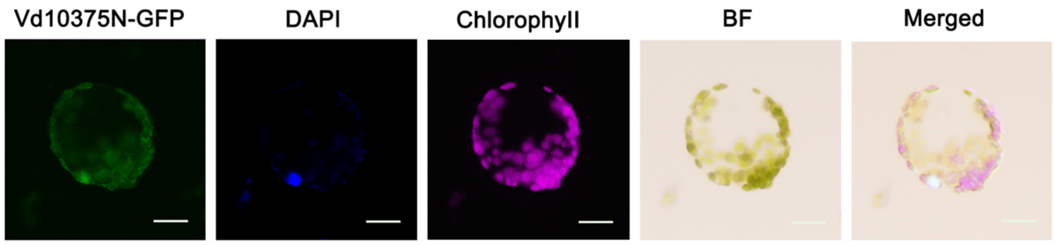

Supplementary Fig. S3. Subcellular localization of Vd10375. Vd10375N-GFP was transiently expressed via *Agrobacterium*-mediated infiltration. Protoplasts were isolated at 48 hpi, and GFP fluorescence was observed using confocal microscopy. Scale bars = 10 μm.

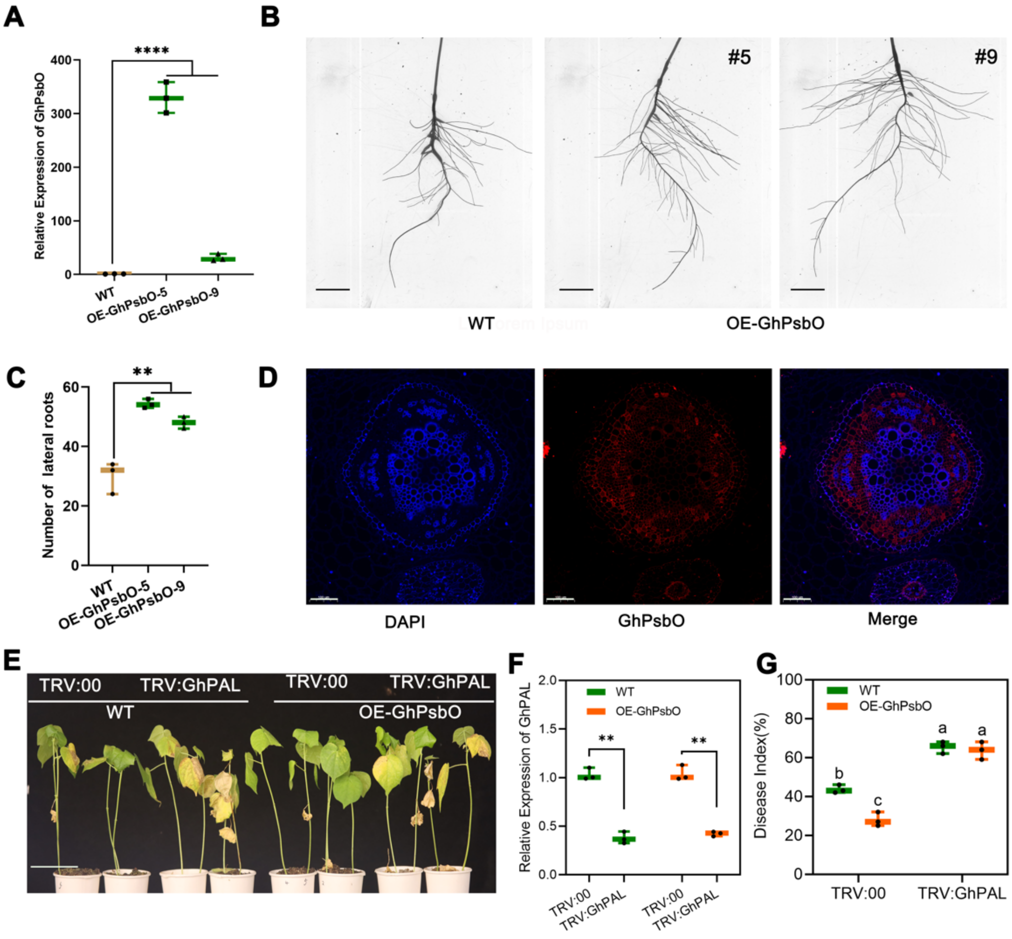

Supplementary Fig. S4. Phenotypic identification of OE-GhPsbO. A. qRT-PCR analysis confirming elevated *GhPsbO* transcript levels in *OE-GhPsbO* lines compared

to WT (*GhUBQ7* as internal control) (Student's *t*-test, ****$P < 0.0001$). Each data point represents the mean of three independent experiments in which each experiment contained three replicates. The error bars represent s.e.m. (n=3). **B**. Root scanning was performed using EPSON Scan software, and the images showed that the OE-GhPsbO line exhibited an increase in root number and biomass compared to the wild-type (WT). Scale bar = 1 cm **C**. Statistical analysis of lateral root number using WinRHIZO software in wild-type and transgenic lines (Student's *t*-test, **$P < 0.01$). Each data point represents the mean of three independent experiments in which each experiment contained three replicates. The error bars represent s.e.m. (n=3). **D**. Gene localization of GhPsbO in cotton root tissues was performed using fluorescence in situ hybridization, with red indicating the gene expression sites, Xy, xylem, and Ph, phloem. Scale bars = 100 μm. **E.** Phenotypes of silencing GhPAL in wild-type and OE-GhPsbO plants. Scale bars = 8 cm. **F-G.** Detection of GhPAL silencing efficiency and statistical analysis of disease index.

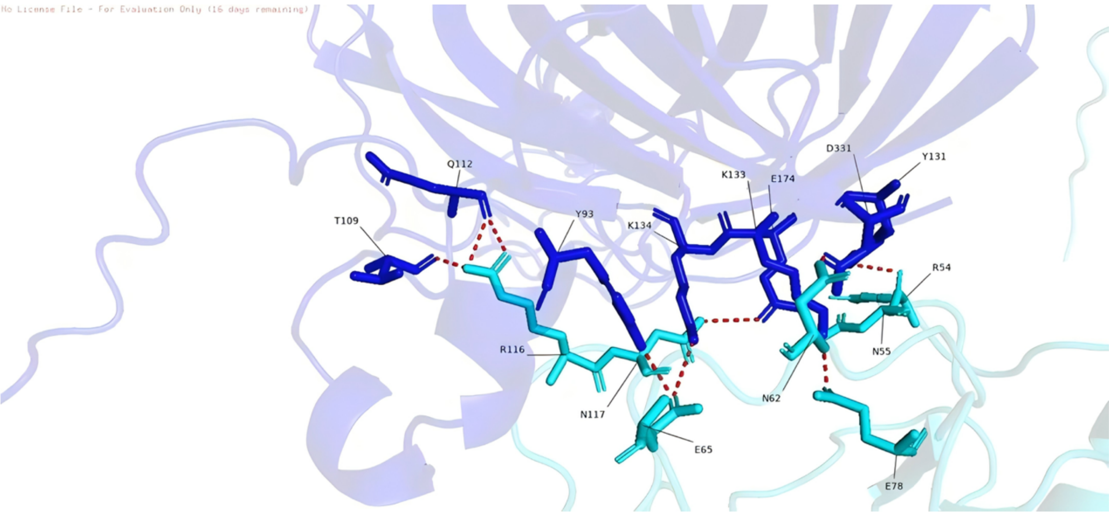

Supplementary Fig. S5. AlphaFold-based molecular docking model illustrating the interaction surface between Vd10375 (cyan) and GhPsbO (blue).

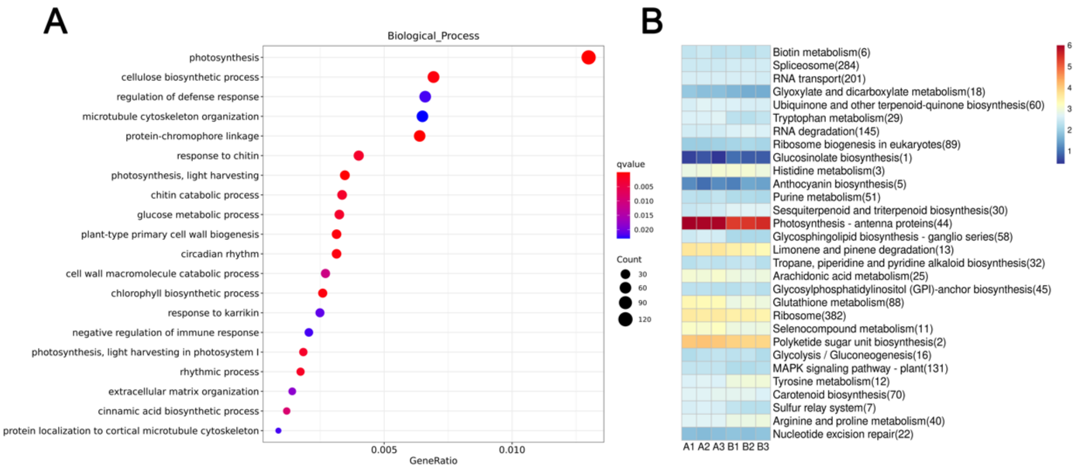

Supplementary Fig. S6. Gene Ontology (GO) (**A**) and KEGG (**B**) pathway enrichment analyses of the target genes of differentially expressed lncRNAs in WT and *OE-Vd10375N* plants.

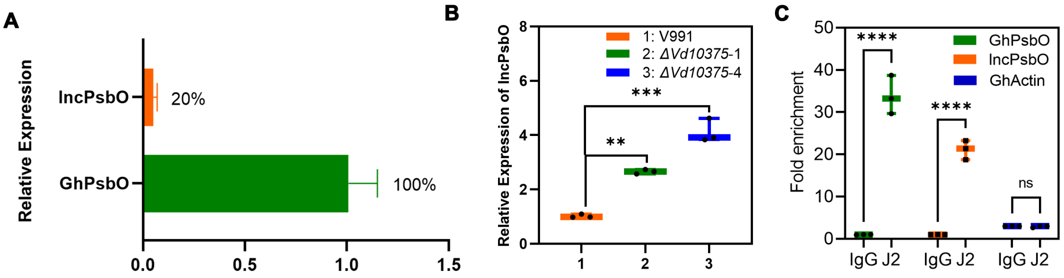

Supplementary Fig. S7. Correlation analysis of lncPsbO. A. Determination of GhPsbO and lncPsbO levels in root tissues of uninfected cotton lines. Each data point represents the mean of three independent experiments in which each experiment contained three replicates. The error bars represent s.e.m. (n=3). B. Quantitative RT-PCR analysis revealed elevated *lncPsbO* transcript levels in cotton roots infected with *ΔVd10375* mutants compared to the wild-type (Student's *t*-test, $^{**}P < 0.01$, $^{***}P < 0.001$). Each data point represents the mean of three independent experiments in which each experiment contained three replicates. The error bars represent s.e.m. (n=3). C. Double-stranded RNA immunoprecipitation (dsRIP) analysis GhPsbO and lncPsbO enrichment following anti-J2 antibody immunoprecipitation, and GhActin was used as a negative control (Student's *t*-test, $^{**}P < 0.01$). Each data point represents the mean of three independent experiments in which each experiment contained three replicates. The error bars represent s.e.m. (n=3).

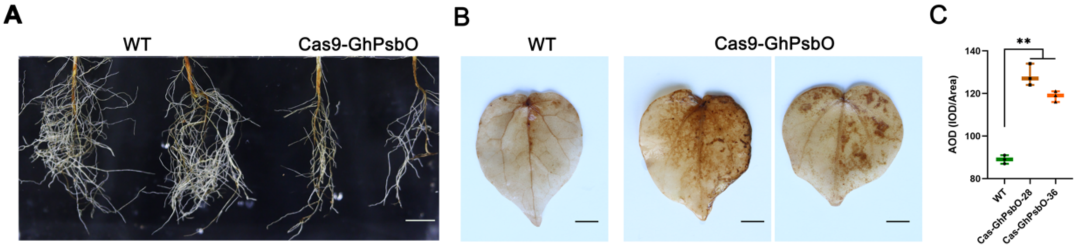

Supplementary Fig. S8. Phenotypic identification of *Cas9-GhPsbO.* **A**. Root morphological analysis revealed reduced root development in *Cas9-GhPsbO* plants compared to WT. Scale bars = 1 cm. **B**. DAB staining of leaves from WT and *Cas9-GhPsbO* plants indicated elevated ROS levels in the knockout lines. Scale bars = 1 cm. **C**. Images obtained after DAB staining were quantified using ImageJ software (Student's *t*-test, $^{**}P < 0.01$). Each data point represents the mean of three independent experiments in which each experiment contained three replicates. The error bars represent s.e.m. (n=3).

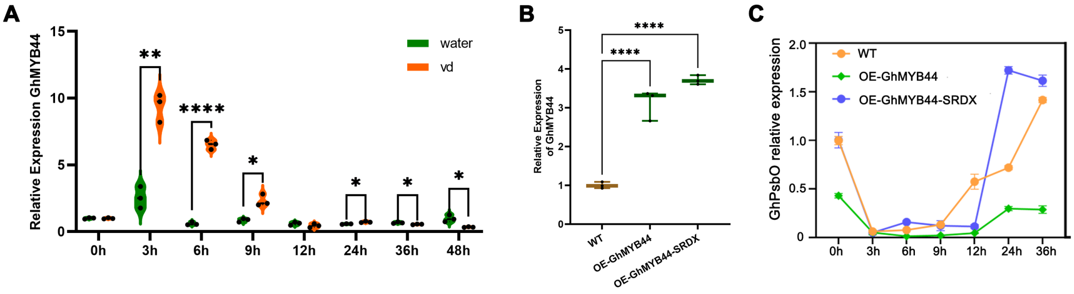

Supplementary Fig. S9. *V. dahliae* induces *GhMYB44* expression. A. Temporal expression profiling of *GhMYB44* during *V. dahliae* infection via qRT-PCR using cotton roots sampled at multiple time points post-inoculation (Student's *t*-test, $^{*}P < 0.05$, $^{**}P < 0.01$, $^{****}P < 0.0001$). Each data point represents the mean of three independent experiments in which each experiment contained three replicates. The error bars represent s.e.m. (n=3). **B**. Expression levels of *GhMYB44* in *OE-GhMYB44* and *OE-GhMYB44-SRDX* transgenic plants were validated by qRT-PCR (Student's *t*-test, $^{****}P < 0.0001$). Each data point represents the mean of three independent experiments in which each experiment contained three replicates. The error bars

represent s.e.m. (n=3). **C**. qRT-PCR analysis showed reduced *GhPsbO* expression in *OE-GhMYB44* lines and increased expression in *OE-GhMYB44-SRDX* lines (a dominant repressor form), under *V. dahliae*-infected conditions, 0 h was used as a mock control. Each data point represents the mean of three independent experiments in which each experiment contained three replicates. The error bars represent s.e.m. (n=3).
